# Single-cell multiome characterizing intercellular communication and intracellular regulation of epithelium and mesenchymal during secondary palate development in mice

**DOI:** 10.1016/j.csbj.2025.09.031

**Published:** 2025-09-24

**Authors:** Usama Hussein, Yulin Dai, Andi Liu, Toshiyuki Itai, Fangfang Yan, Lukas M. Simon, Zhongming Zhao

**Affiliations:** a Center for Precision Health, McWilliams School of Biomedical Informatics, The University of Texas Health Science Center at Houston, Houston, TX 77030, USA; b Department of Epidemiology, Human Genetics and Environmental Sciences, School of Public Health, The University of Texas Health Science Center at Houston, Houston, TX 77030, USA; c Therapeutic Innovation Center, Baylor College of Medicine, Houston, TX 77030, USA

**Keywords:** Cellular communication, Gene regulatory network, Palatogenesis, Signaling pathway, Single-cell multi-omics

## Abstract

Cell-cell communication between adjacent tissues is fundamental to orchestrating organ morphogenesis. During secondary palate development, interactions between epithelial and mesenchymal cells regulate epithelial-mesenchymal signaling, thereby controlling cell proliferation, differentiation, and tissue patterning. However, the precise molecular mechanisms underlying these interactions remain largely unexplored. In this study, we employed single-cell multiome sequencing to simultaneously profile chromatin accessibility and gene expression in 35,150 individual cells isolated from the developing mouse secondary palate at embryonic days E12.5, E13.5, E14.0, and E14.5. Our analysis revealed that intercellular signaling plays a pivotal role in directing secondary palate morphogenesis, with WNT, BMP, and PDGF pathways emerging as key regulators of this process. These pathways exhibit peak activity at E12.5, followed by a progressive decline toward E14.5. Notably, WNT signaling is predominantly enriched in the nasal epithelium, mediating interactions with neighboring cell populations, while BMP signaling is more prominent in the oral epithelium. Additionally, PDGF signaling exhibits enhanced activity in the dental epithelium, indicating its role in coordinating epithelial-mesenchymal interactions during palatal development. Collectively, these findings provide novel insights into the spatiotemporal associations of WNT, BMP, and PDGF signaling with intercellular communication during secondary palate development, suggesting that these pathways may contribute to the cellular dynamics underlying secondary palate morphogenesis. Moreover, they underscore the need for further investigation.

## Graphical Abstract

**Figure:**
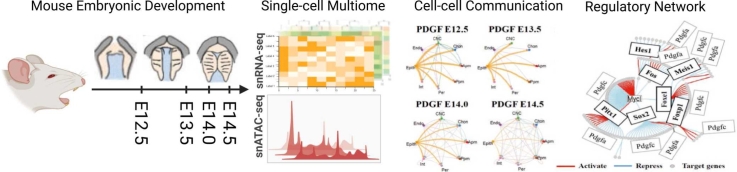

## 1 Introduction

Secondary palate development initiates around E11.5 in mice. During this process, the maxillary processes on the oral side begin to grow downwards, giving rise to paired palatal shelves, while the tongue develops from the mandible [1]. At E12 to E13.5 in mouse embryos, the palatal shelves undergo vertical growth, flanking the developing tongue. Subsequently, from E13.5 to E14.5, the bilateral palatal shelves reposition themselves horizontally above the tongue and grow towards the midline. This pattern is followed by palatal fusion, then culminates in the formation of the roof of the oral cavity. The palatal fusion is typically completed by the 12th week of human gestation and by approximately E16.5 in mice [1], [2]. Extensive wet lab research has elucidated the involvement of major signaling pathways and gene regulatory networks to investigate the molecular mechanism controlling palatal shelf growth, patterning, and fusion [1], [3].

Epithelial tissues play a vital role in driving morphogenesis [4], [5], [6]. They serve as functional barriers, separating the internal structures of organs from the external environment. Nonetheless, there is still a lack of comprehensive understanding regarding how organs precisely regulate their shape during specific developmental stages. The development of the upper lip and palate depends on the coordinated growth and pattern formation of facial structures, as well as the timely merging and adhesion of distinct primordia. This intricate process makes facial development vulnerable to any deficiency issues of the outer layer of epithelial cell integrity [7]. Apart from serving as the structural basis for controlling the timing and location of various signaling molecules that guide the growth and patterning of facial structures, the integrity of the epithelium plays a vital role in generating biomechanical forces within the surface layer of epithelial cells. These forces help push the maxillary and lateral nasal processes toward the midline, where they can establish contact and fusion with the medial nasal process. This fusion process fills the groove between the two medial nasal processes [1], [2], [3].

Recently, single-cell multi-omics technologies have proven highly effective for understanding the regulation of genes at the level of cellular dynamics, as they enable simultaneous profiling of gene expression and chromatin accessibility within individual cells. Although single-cell RNA sequencing (scRNA-seq) has revolutionized our knowledge of cellular heterogeneity and differentiation trajectories [8], [9], it has inherent limitations in directly assessing the mechanisms of intercellular communication. Nevertheless, because scRNA-seq data provide quantitative gene expression profiles, they can still be used through computational inference methods to approximate potential ligand-receptor interactions and signaling networks that underlie cell-cell communication [10], [11]. In this context, multi-omics approaches offer complementary layers of information that strengthen such inferences and provide a more comprehensive understanding.

Here, we utilized our in-house single-cell multiome sequencing dataset from the developing mouse secondary palate at four distinct developmental stages to conduct a comprehensive analysis of cell-cell communication. Previously, we characterized broad transcriptional and chromatin accessibility landscapes. In this work, we aim to further uncover the dynamic roles of regulatory signaling in secondary palate development, highlighting their contributions to epithelial-mesenchymal interactions and cell movement during palatogenesis. Through our intercellular communication analysis, we aimed to infer, visualize, and quantify the probabilities of signaling interactions between the 8–10 major cell types identified across different developmental stages. Given prior evidence that epithelial cells play a central signaling role during palatal shelf elevation and medial edge seam formation in initiating palatal fusion [12], [13] and consistent with our dataset showing prominent epithelial signaling activity at E12.5, we specifically focused on this population to characterize its regulatory role in secondary palate development.

## 2 Results

### 2.1 Processing single-cell multiomics assay in secondary palate development

Our goal is to examine the intercellular communication patterns along with gene regulatory networks among different cell populations within the secondary palate during these critical time points. To investigate this, the study adopted single-cell multiomics sequencing analysis of published data with GEO accession number (GSE218576), which included samples collected at E12.5 (n = 2), E13.5 (n = 3), E14.0 (n = 2), and E14.5 (n = 2) to capture the gene expression patterns at the cellular level in the secondary palate. Following the previous work [14], we applied filtering criteria to both assays and conducted integration analysis using R package Seurat v4. This process identified a total of 35,150 cells with high-quality measurements across 35,160 genes and 123,749 accessible peaks, representing potential cis-regulatory elements. The filtered dataset was then used for downstream analyses, including the inference of gene regulatory networks and cell-cell communication (Supplementary Figure S1).

**Fig. 1:**
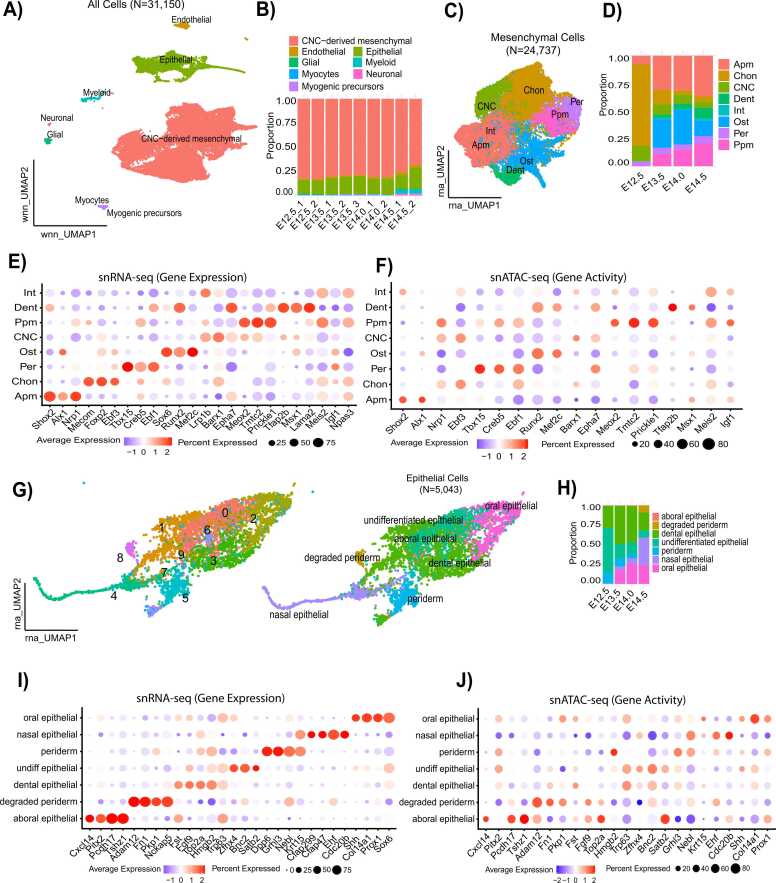
Single-cell multiome assays were employed to analyze changes in both transcriptome and epigenome during the embryonic stages of the mouse secondary palate. (A) UMAP visualization of 35,150 cells based on Weighted Nearest Neighbor (WNN) analysis, with each data point representing an individual cell. (B) Stacked bar plot showing the frequencies of major cell types in each sample, with different colors indicating distinct cell types. (C) Re-clustered subset of CNC-derived mesenchymal cells, illustrating the mesenchymal cell subtypes. (D) Stacked bar plot depicting the frequencies of CNC-mesenchymal subtypes across different developmental stages. (E, F) Dot plots presenting marker gene expression and gene activity across various CNC-mesenchymal cell subtypes. Dot size indicates the percentage of cells expressing the gene, with colors representing expression levels from low (grey) to high (blue). (G) Re-clustered subset of epithelial cells, showing various epithelial subtypes. (H) Stacked bar plot displaying the frequencies of epithelial subtypes across developmental stages. (I, J) Dot plots presenting marker gene expression and gene activity across various epithelial cell subtypes. E12.5, E13.5, E14.0, E14.5: the 'E' refers to embryonic days in mouse development.

We identified 16 distinct clusters identifiable as 16 cell types/or subtypes in both transcriptome and open-chromatin data using the known genetic markers (Supplementary Figures S2A and S2B). We carried out an unsupervised framework ‘weighted-nearest neighbor’ (WNN) in our analysis to allow the assessment of the relative usefulness of each data type in each cell, facilitating the integrative analysis of multiple modalities [15] (Fig. 1A and Supplementary Figures S2A and S2B). The major cells were defined by canonical genetic markers according to the cellular annotations in our work of the original published paper [14]: Cranial neural crest (CNC)-derived mesenchymal cells (Lrp1b+, Pax9+, Twist1), myocytes (Myh3+, Myod1+, Myog+), myogenic progenitor cells (Fgfr4+, Pax7+, Myf5+), endothelial cells (Zfpm2+, Cdh5+, Cldn5+), myeloid cells (Arhgap15+, Lyz2+, Ptprc+), neurons (Stmn2+, Tubb3+, Alk+), glial cells (Sox10+, Sntb1+, Slc35f1+) and epithelial cells (Wnt4+, Epcam+, Sox2+). The distribution of prominent cell types within the distinct samples at varying developmental stages reveals that among all cell types, CNC-derived mesenchymal and epithelial cells comprise the largest proportion (Fig. 1B). To validate our findings prior to E12.5, we analyzed a published dataset from embryonic stage E11.5 retrieved from the published article with accession number GSE132462 [16]. The clustering pattern was consistent with our results, with mesenchymal cells comprising the largest population, followed by epithelial cells (Supplementary Figure S2C).

Within the analyzed samples, certain lineages exhibited multiple clusters, highlighting cellular heterogeneity within these populations, particularly among CNC-derived mesenchymal cells (Fig. 1A and C). Notably, eight distinct subclusters were identified within the CNC-derived mesenchymal population: CNC, chondrogenic (Chon), perimysial (Per), posterior palatal mesenchymal (Ppm), anterior palatal mesenchymal (Apm), intermediate mesenchymal (Int), osteogenic (Ost), and dental mesenchymal (Dent), as detailed in Fig. 1C. Our investigation of mesenchymal dynamics across developmental stages indicated a progressive increase in the fraction of anterior and posterior palatal mesenchymal cells from E12.5 to E14. In contrast, the populations of CNC-derived progenitor cells and chondrocytes declined from E12.5 to E14.5 (Fig. 1D). To further characterize these subpopulations, we identified the top differentially expressed genes and analyzed gene activity scores, integrating accessible chromatin regions overlapping gene bodies and promoters in the snATAC-seq dataset. The cell-type annotations were manually curated based on highly specific markers identified in the original published paper [14] (Fig. 1E and F).

We subsequently subsetted and re-clustered the epithelial cells, identifying six distinct subtypes: aboral epithelial cells ("aboral epith"), dental epithelial cells ("dent epith"), nasal epithelial cells ("nasal epith"), periderm, undifferentiated epithelial cells ("undiff epith"), and oral epithelial cells ("oral epith"). These subtypes were annotated based on established marker genes from previous studies, as referenced in the previously published work [14] (Fig. 1G and I).

To investigate the roles of these subpopulations, we examined the most differentially expressed genes within each cluster (Fig. 1I). Analysis of the distribution of key epithelial populations across embryonic stages revealed a gradual increase in the proportion of aboral, nasal, and oral epithelial cells from E12.5 to E14.5. In contrast, periderm cells decreased in proportion between E12.5 and E14.5 (Fig. 1H). Additionally, we assessed gene activity scores, which were derived by integrating accessible chromatin regions overlapping both gene bodies and promoters in the snATAC-seq dataset (Fig. 1J).

### 2.2 Epithelial cell populations exhibit distinct signaling patterns

Considering the distinct spatial organization of epithelial and mesenchymal cell clusters within the developing secondary palate, as well as their temporal progression across embryonic stages, we hypothesized that these populations exhibit functional differences in their intercellular signaling across the four developmental stages. To explore this, we utilized CellChat cellular communication analysis to systematically examine intercellular signaling dynamics [17].

Our single-cell multiome dataset was clustered into eight major cell groups (Fig. 1A). To robustly characterize intercellular signaling dynamics, we adopted a two-step, hypothesis-driven workflow. First, we compared the eight mesenchymal subclusters (Fig. 1C) (and endothelial cells) to the entire epithelial population to detect strong, stage-dependent epithelial-mesenchyme signaling while maximizing statistical power. This coarse-grained comparison minimizes noise from small subpopulations and identifies developmental stages with the most pronounced communication interaction strength changes (here, a peak at E12.5), contrasting with the trends observed in mesenchymal and epithelial populations (Supplementary Figure S3A). Second, after establishing the timing and directionality of these global signals, we reclustered epithelial cells into six subpopulations (Fig. 1G) and performed targeted subcluster-level communication analyses to localize which epithelial subsets drive the observed signals. Further CellChat analysis revealed that dental, nasal, and oral epithelial populations exhibited a progressive reduction in interaction strength over time (Supplementary Figure S3B). In contrast, the periderm, undifferentiated epithelial, and aboral epithelial subpopulations exhibited weaker and less consistent interaction patterns, with no strong enrichment of canonical signaling pathways such as WNT, BMP, or PDGF (Supplementary Figure S3C).

**Fig. 2:**
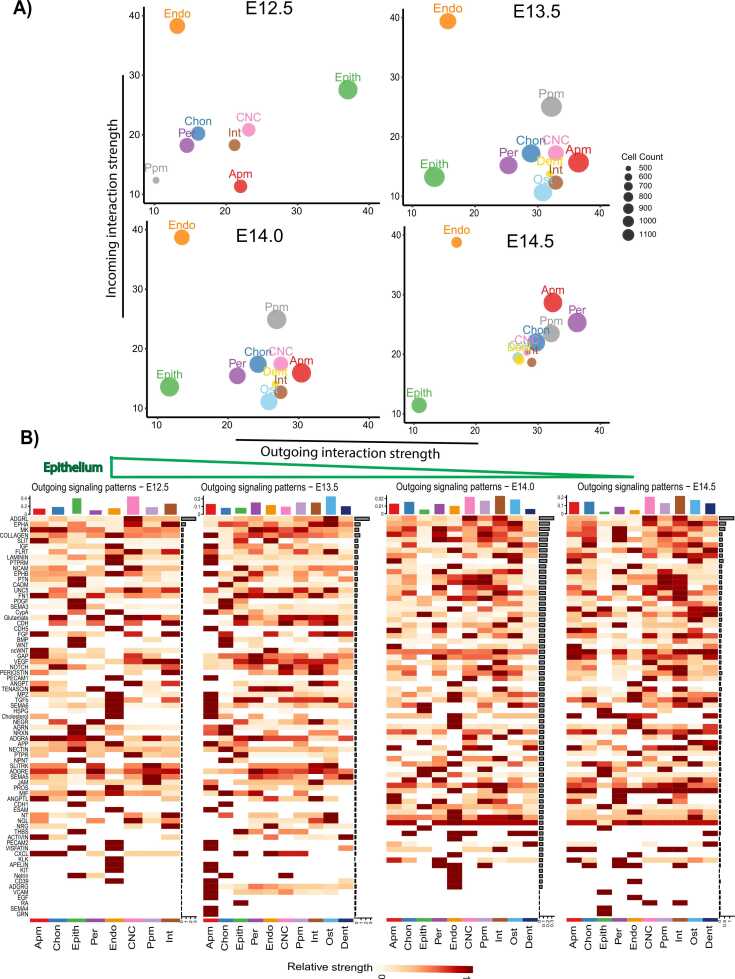
Unsupervised clustering of the major sources and targets from the interaction strength in two dimensions. (A) Joint projection and clustering of prominent cells from different developmental stages onto a two-dimensional space, categorized by their roles in sending or receiving signaling networks. The figure highlights epithelial cells as a significant source and target of signals directed toward neighboring cells. Dot size corresponds to the number of expressed cells, and colors denote cell types. (B) Heatmap illustrating outbound signaling pathways from major cell types across various developmental stages. The top bar plot indicates the proportion of cell types involved in sending signals to neighboring cells. The horizontal green arrow signifies epithelial cells at E12.5 as primary signal sources compared to the other embryonic stages. The stacked bar plots flanking the heatmap correspond to the y-axis, representing distinct signaling pathways between cell types (x-axis).

Unsupervised clustering in 2D space (Fig. 2A, B) enabled a comparative assessment of the outgoing and incoming signaling interactions across developmental stages. Scatter plots and heatmap analyses demonstrated that epithelial cells were the predominant sources and recipients of signaling at E12.5, whereas mesenchymal populations assumed this role by E14.5. Analysis of epithelial subpopulations revealed that dental, nasal, and oral epithelial cells exhibited the highest outgoing signaling activity at E12.5, which progressively decreased over developmental time (Supplementary Figure S3B). The remaining subpopulations periderm, undifferentiated epithelial, and aboral epithelium, showed relatively weak and less consistent interaction patterns, with minimal enrichment of canonical signaling pathways such as WNT, BMP, or PDGF (Supplementary Figure S3C). These results indicate that the overall epithelial signaling trend during secondary palate development is largely driven by the dental, nasal, and oral epithelial subpopulations.

CellChat analysis predicted 161 significant ligand-receptor interactions within 35 signaling pathways at E12.5, including BMP, WNT, ncWNT, TGFβ, NGF, FGF, PDGF, NPNT, NRXN, and SEMA3/4/5/6. At E13.5, 147 interactions were detected within 35 pathways, including TGFβ, BMP, WNT, ncWNT, PDGF, IGF, SEMA3/4/6, LAMININ, COLLAGEN, and AGRN. By E14.0, 138 interactions were identified within 34 pathways, involving TGFβ, BMP, WNT (Wnt2/4/2b/7b), ncWNT, EGF, PDGF, PTN, CDH, NOTCH, and SEMA3/4/6. At E14.5, 145 interactions persisted within 34 pathways, including BMP, WNT (Wnt4/6/7b), ncWNT, FGF, PDGF, IGF, MK, PTN, and SEMA3/6 (Fig. 2B).

Unsupervised clustering further confirmed epithelial cells as key signaling mediators (Fig. 2A). At E12.5, dental, nasal, and oral epithelial cells exhibited enriched signaling pathways, including CADM, PTN, PDGF, SEMA3, AGRN, MIF, NRXN, and VISFATIN in dental epithelium; MK, WNT, AGRN, NECTIN, NPNT, EGF, and GRN in nasal epithelium; and CADM, SEMA3, BMP, HH, THBS, PROS, and NGF in oral epithelium (Supplementary Figure S4B). These findings underscore the dynamic associations of epithelial signaling during palatogenesis and suggest that epithelial cells may play important roles in intercellular communication during early palate development, as inferred from the strength and enrichment of outgoing signaling pathways at E12.5.

### 2.3 WNT pathway as a key signaling source in epithelial cells

**Fig. 3:**
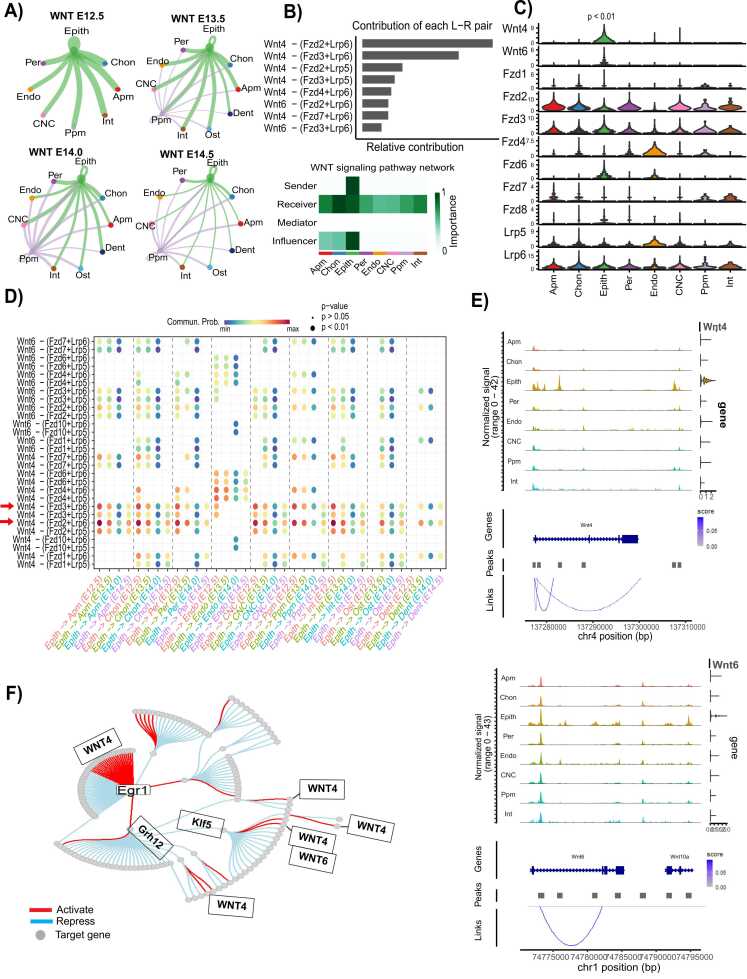
CellChat analysis of the WNT signaling communication network in epithelial cells across different developmental stages. (A) Hierarchical plots depict the inferred intercellular communication network for WNT signaling. Circle sizes are proportionate to the number of cells in each cell type, and edge width represents communication strength probability. (B) (Top) The relative contribution of each ligand-receptor pair to the overall communication network of the WNT signaling pathway is calculated as the ratio of the total communication probability of the inferred network of each L-R pair to that of the WNT signaling pathway. (Bottom) The WNT signaling network shows that the epithelial cell is the major contributor in the network. (C) Violin plot displaying the expression distribution of signaling genes involved in the inferred WNT signaling network. (D) The dot plot illustrates the relative communication probability of the contributing L-R pair from epithelial cells to neighboring mesenchymal cells. Dot size signifies the statistical significance (P < 0.05) of the communication probability, and color indicates communication probability strength. (E) Top: Genome Browser visualization of aggregated chromatin accessibility at the chr4: 137.28–137.31 (Mb) locus for each major cell type, alongside *Wnt4* gene expression. Arcs at the bottom represent positively linked CRE-Wnt4 pairs. The linkage between Wnt4 and chr4:137280000–137310000 is highlighted within a blue box. Bottom: Genome Browser visualization of aggregated chromatin accessibility at the chr1: 74.775–74.795 (Mb) locus for each major cell type, coupled with *Wnt6* gene expression. Arcs at the bottom indicate positively linked CRE-Wnt6 pairs. The linkage between Wnt6 and chr1: 74775000–74795000 is highlighted within a blue box. (F) Retrieved gene regulatory network (GRN) of epithelial cells, showcasing Wnt4/6 regulation by a set of transcription factors. Color indicates repression (blue) or activation (red) of target genes (grey circle).

Epithelial cells serve as the primary contributors to ligand signaling, with their signaling activity peaking at E12.5 before declining by E14.5 (Fig. 3A). Within this communication network, the WNT signaling pathway plays a central role, primarily driven by the WNT ligand-receptor interactions Wnt4-(Fzd2+Lrp6) and Wnt4-(Fzd3+Lrp6) (Fig. 3B). At E12.5, epithelial cells exhibit significantly higher communication probabilities with mesenchymal populations compared to later developmental stages (Figs. 3A, C). This heightened signaling activity is characterized by the elevated expression of Wnt4 and Wnt6, which distinguishes epithelial cells at this stage (Fig. 3C) and showed an elevation of communication probability for Wnt ligand-receptor pathway (Fig. 3D). Furthermore, chromatin accessibility analysis reveals the association of these genes with prominent chromatin aggregation and ATAC peak enrichment, supporting their regulatory role in epithelial-mesenchymal interactions during palatogenesis (Fig. 3E). Gene regulatory network analysis further demonstrated that Wnt4 and Wnt6 expression are enhanced by transcription factors Klf1/5 and Egr1 (Fig. 3F). A detailed cell-cell communication analysis revealed that the nasal epithelium serves as the primary source of WNT signaling to mesenchymal populations at E12.5, distinguishing this developmental stage from others (Supplementary Figure S5A and S5B). Our analysis shows that this communication network is primarily driven by Wnt4 signaling via the Fzd2+Lrp6 and Fzd3+Lrp6 receptor complexes (Supplementary Figure S5C). Moreover, nasal epithelial cells at E12.5 exhibit significantly higher communication probabilities with mesenchymal populations, which further supports this observation (Supplementary Figure S5E). The enriched expression of Wnt4 within the nasal epithelium reinforces its critical role in facilitating epithelial-mesenchymal interactions (Supplementary Figure S5D). Notably, Wnt4 signaling via the Fzd2+Lrp6 and Fzd3+Lrp6 receptor complexes demonstrates high communication probabilities, emphasizing its dominant role in epithelial-derived WNT signaling (Fig. 3B, Supplementary Figure S5C).

### 2.4 The BMP pathway as a principal signaling mechanism in epithelial cells at E12.5

**Fig. 4:**
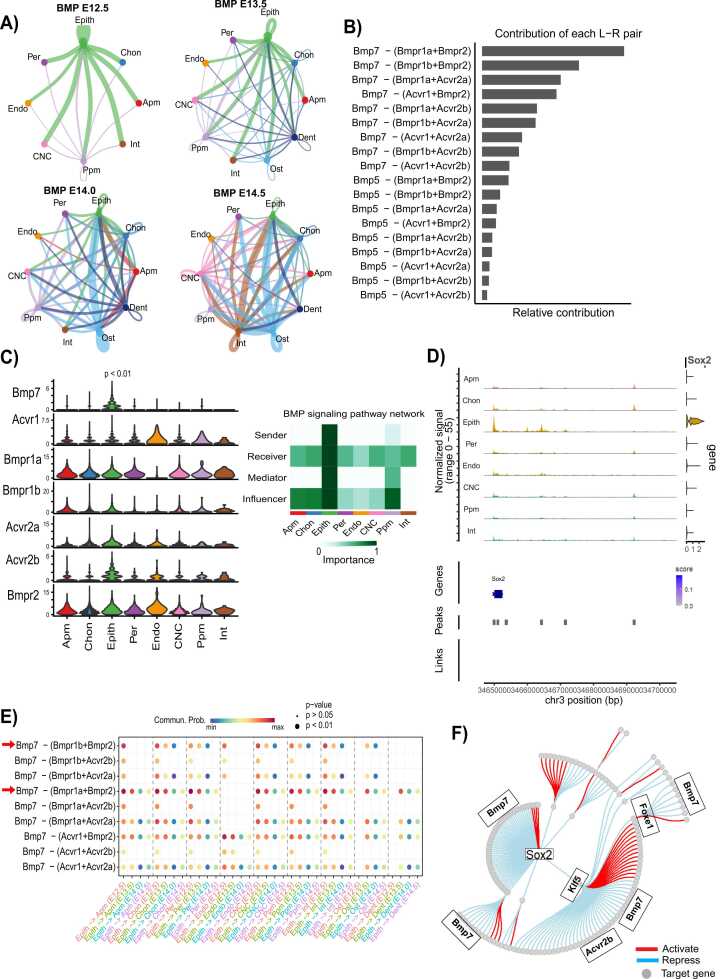
Analysis of BMP signaling network in epithelial cells at various developmental stages using CellChat. (A) Hierarchical plots display the inferred intercellular communication network for BMP signaling. The left segment highlights autocrine and paracrine signaling to epithelial cell states, while the right segment focuses on other mesenchymal cell states. Circle sizes correspond to the number of cells in each type, and edge widths represent communication probability. (B) The relative contribution of each ligand-receptor pair to the overall BMP signaling network is shown as the ratio of the total communication probability of each pair to the pathway's total. (C) (Left) A violin plot presents the expression distribution of signaling genes enriched in the BMP signaling network. (Right) The BMP signaling network shows that the epithelial cell is the major contributor in the network. (D) The top section shows a Genome Browser visualization of aggregated chromatin accessibility at chr3: 34.652–34.660 (Mb) locus for each major cell type, alongside *Sox2* gene expression (a regulator of Bmp7). Arcs at the bottom represent positively linked CRE-Sox2 pairs, with the Sox2 and chr3: 34652000–34660000 linkage highlighted in blue. (E) A dot plot depicts the relative communication probability of ligand-receptor pairs from epithelial cells to mesenchymal cells. Dot size indicates the statistical significance (P < 0.05) of communication probability, and color represents its strength. (F) The retrieved gene regulatory network (GRN) of epithelial cells shows Bmp7 regulation by various transcription factors. Colors indicate repression (blue) or activation (red) of target genes (grey circles).

CellChat analysis identified a highly significant BMP signaling subnetwork, highlighting the pivotal role of epithelial cells in initiating ligand signaling to mesenchymal subpopulations, particularly at E12.5, with a subsequent decline by E14.5 (Fig. 4A). The primary ligand-receptor interactions driving this communication include Bmp7-(Bmpr1a+Bmpr2) and Bmp7-(Bmpr1b+Bmpr2) (Fig. 4B). At E12.5, epithelial cells exhibit markedly higher communication probabilities with mesenchymal populations compared to later stages (Fig. 4E), a finding further supported by the elevated expression of Bmp7 in epithelial cells (Fig. 4C).

A more detailed investigation using cell-cell communication analysis revealed that the oral epithelium serves as the predominant source of BMP signaling to mesenchymal populations at E12.5, distinguishing this stage from others (Supplementary Figures S6A and S6B). The communication network is primarily driven by Bmp7-(Bmpr1a+Bmpr2) and Bmp7-(Bmpr1b+Bmpr2) (Supplementary Figure S6C). Consistently, oral epithelial cells at E12.5 demonstrate significantly higher communication probabilities with mesenchymal populations, corroborating these results (Supplementary Figure S6E). The enriched expression of BMP7 in the oral epithelium further substantiates its role in mediating epithelial-mesenchymal interactions (Supplementary Figure S6D).

Additionally, gene regulatory network analysis indicates that Bmp7 activity is modulated by transcription factors Sox2, Pitx1, Foxe1, and Klf5 (Fig. 4F). Notably, Sox2 is closely associated with its ATAC peaks and chromatin aggregation in epithelial cells, reinforcing its regulatory influence on BMP signaling (Fig. 4D). Between the two primary signaling interactions, Bmp7-(Bmpr1a+Bmpr2) exhibits higher communication probabilities than Bmp7-(Bmpr1b+Bmpr2) (Fig. 4E, Supplementary Figure S6E), underscoring its dominant role in epithelial-mediated BMP signaling.

### 2.5 The PDGF pathway as a principal signaling mechanism in epithelial cells

**Fig. 5:**
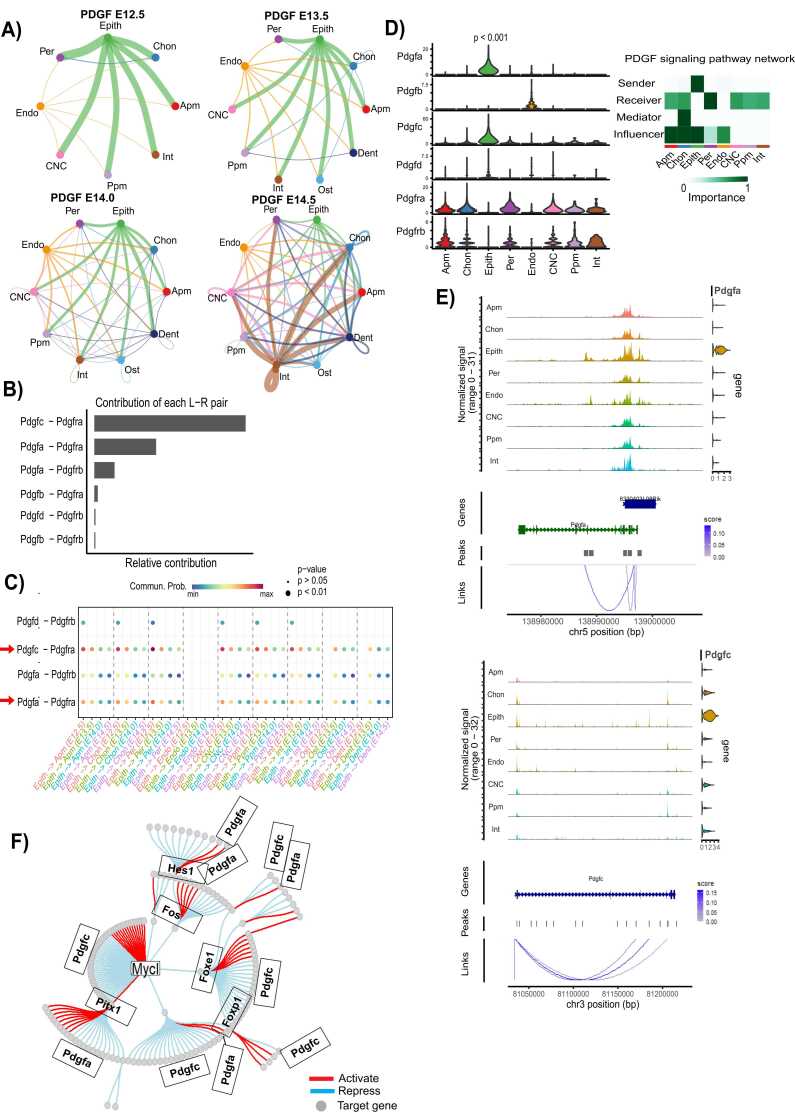
Analysis of PDGF signaling network in epithelial cells across diverse developmental stages using CellChat. (A) Hierarchical plots depict the inferred intercellular communication network for PDGF signaling. The left side illustrates autocrine signaling to epithelial cell states, and the right side illustrates paracrine signaling to other mesenchymal cell states. Circle sizes are proportional to the number of cells in each type, and edge widths represent communication probabilities. (B) The relative contribution of each ligand-receptor pair to the overall PDGF signaling network is calculated as the ratio of the total communication probability of each pair to the entire pathway. (C) Dot plot showcasing the relative communication probability of ligand-receptor pairs from epithelial cells to mesenchymal cells. Dot size indicates the statistical significance (P < 0.05) of communication probability, and color represents its strength. (D) (Left) Violin plot displaying the expression distribution of signaling genes enriched in the PDGF signaling network. (Right) The BMP signaling network showing the epithelial cell is the major contributor in the network. (E) Top: Genome Browser visualization of aggregated chromatin accessibility at the chr5: 138.98–139.00 (Mb) locus for each major cell type, along with *Pdgfa* gene expression. Arcs at the bottom represent positively linked CRE-Pdgfa pairs, with the linkage highlighted in blue. Bottom: Genome Browser visualization of aggregated chromatin accessibility at the chr3: 81.050–81.200 (Mb) locus for each major cell type, along with *Pdgfc* gene expression. Arcs at the bottom represent positively linked CRE-Pdgfc pairs, with the linkage highlighted in blue. (F) Retrieved gene regulatory network (GRN) of epithelial cells showing Pdgfa and Pdgfc regulation by various transcription factors. Colors indicate repression (blue) or activation (red) of target genes (grey circles).

Cell-cell communication analysis revealed a significant PDGF signaling network, highlighting epithelial cells as the predominant source of ligand signaling to neighboring mesenchymal populations, particularly at E12.5, with a progressive decline by E14.5 (Fig. 5A). The primary ligand-receptor interactions driving this communication are Pdgfc-Pdgfra and Pdgfa-Pdgfra (Fig. 5B). At E12.5, epithelial cells exhibit markedly elevated communication probabilities with mesenchymal populations compared to later stages (Fig. 5C). This finding is further supported by the increased expression levels of Pdgfa and Pdgfc in epithelial cells (Fig. 5D), with significant chromatin aggregation at their respective ATAC peaks, indicating transcriptional regulation (Fig. 5E).

A more detailed examination using CellChat analysis identified dental epithelium as the principal PDGF signaling source at E12.5, a pattern distinct from later embryonic stages. The Pdgfc-Pdgfra and Pdgfa-Pdgfra ligand-receptor pairs drive this network, with pronounced communication probabilities from dental epithelial cells to mesenchymal and endothelial cells (Supplementary Figures S7A–S7E). Gene regulatory network analysis further demonstrated that Pdgfa expression is enhanced by transcription factors Fos, Hes, Meis1, and Sox2, while being repressed by Pitx1. In contrast, Pdgfc expression is repressed by Foxp1, Foxe1, Mycl, and Sox2, but activated by Meis1 (Fig. 5F). Notably, Pdgfc-Pdgfra exhibits higher communication probabilities than Pdgfa-Pdgfra, underscoring its dominant role in epithelial-derived PDGF signaling (Fig. 5C, Supplementary Figure S7E).

## 3 Discussion

Cell-cell communication between adjacent tissues is crucial for orchestrating organ development. In secondary palate formation, interactions between epithelial and mesenchymal cells regulate cell proliferation and differentiation, shaping craniofacial development. However, the precise molecular mechanisms governing these interactions remain unclear. In this study, we leveraged an in-house single-cell multiome sequencing dataset to simultaneously assess chromatin accessibility and gene expression in 35,150 cells from the mouse secondary palate across embryonic days E12.5, E13.5, E14.0, and E14.5. Our analysis revealed that WNT, BMP, and PDGF signaling pathways play pivotal roles in secondary palate development, with their activity peaking at E12.5 and progressively declining by E14.5.

Secondary palate formation is a highly coordinated developmental process involving the reorientation and outgrowth of the palatal shelves [18]. These structures consist of mesenchymal tissue enveloped by an outer epithelial layer, and the interplay between these two compartments is critical for regulating cell proliferation, differentiation, and morphogenesis during craniofacial development. Epithelial-mesenchymal interactions facilitate the transition of epithelial cells into a mesenchymal state within the palatal shelves, a process essential for proper palatogenesis [2], [19], [20], [21]. Recent single-cell transcriptomic profiling of mouse maxillary development (E10.5–E14.5) has provided a complementary perspective on the transcriptional regulatory networks guiding craniofacial morphogenesis [22]. That study resolved mesenchymal fate trajectories and demonstrated that the segregation of dental and palatal mesenchyme emerges as early as E11.5. In addition, key transcription factors directing these lineage transitions were identified, highlighting the importance of gene regulatory networks in establishing the cellular context for subsequent signaling events. Our multiomic analysis builds on this framework by connecting such transcriptional programs with pathway-level regulation, thereby linking early mesenchymal fate specifications to intercellular communication during palate morphogenesis. To gain earlier developmental insight, we analyzed scRNA-seq samples from mouse palate at E11.5. The resulting cell clustering recapitulated the same patterns observed in our multiomic clusters.

In this study, we identify WNT signaling as a key regulatory pathway at multiple stages of epithelial cell development in the mouse secondary palate. Our analysis reveals two primary signaling axes within this communication network: Wnt4-(Fzd2+Lrp6) and Wnt4-(Fzd3+Lrp6). The strong interaction between the nasal epithelium and adjacent mesenchymal populations is supported by the elevated expression of Wnt4, suggesting a highly active WNT signaling environment. This observation is consistent with previous findings demonstrating widespread WNT pathway activation across multiple tissues during craniofacial development [23]. The WNT signaling cascade is initiated through the binding of secreted WNT ligands to a complex receptor system comprising Frizzled (FZD) receptors and low-density lipoprotein receptor-related protein (LRP) co-receptors, which mediate distinct downstream signaling pathways critical for tissue patterning and morphogenesis. WNT signaling, particularly WNT/β-catenin, regulates epithelial-mesenchymal crosstalk, ensuring proper palatal shelf elevation and fusion. Mutations in WNT-related genes have been linked to clefting disorders. PDGF signaling is essential for mesenchymal cell survival and migration, particularly in neural crest-derived mesenchyme, which contributes to palate formation. PDGF deficiency results in defective palatal rugae patterning and fusion failure [24], [25].

Our findings demonstrate significant epithelial-mesenchyme interactions at embryonic day E12.5, predominantly mediated by WNT signaling through FZD2 receptors and LRP6 co-receptors. This highlights the crucial role of FZD2 and LRP6 in developmental disorders such as orofacial clefts [26] and tooth agenesis [24], [25], suggesting that disruptions in LRP6-mediated canonical WNT signaling contribute to the etiology of cleft lip and palate. Our findings complement prior observations of Wnt modulators such as *Dkk1* and *Dkk2* in cleft palate models, reinforcing the importance of Wnt signaling in palatal morphogenesis. By integrating multiomic analyses, we provide additional evidence that Wnt pathway activity is tightly associated with epithelial-mesenchymal interactions during secondary palate development. These results highlight how Wnt modulators and their spatiotemporal regulation may contribute to disrupted osteogenic programming underlying cleft palate pathogenesis [27]. Among the key WNT ligands, Wnt4 and Wnt6 exhibit elevated expression in epithelial cells and the nasal epithelium, consistent with their previously reported roles in the developing palatal primordia during palatogenesis [28]. Notably, Wnt6 expression peaks in epithelial cells at E12.5 and declines at later stages, a pattern that may reflect its role in regulating palatal shelf elongation and elevation. This function is likely mediated through β-catenin signaling, which promotes cell proliferation within the palatal mesenchymal [29]. Overall, these findings underscore the importance of precise spatiotemporal regulation of WNT signaling during craniofacial development and highlight the complex regulatory mechanisms governing lip and palate formation.

Bone morphogenetic proteins (BMPs) are essential morphogens that regulate organogenesis and ossification [30]. BMP signaling is crucial for palatal shelf growth and fusion by controlling mesenchymal proliferation and epithelial-mesenchymal interactions. Disruptions in BMP signaling often lead to cleft palate due to impaired mesenchymal expansion. When BMP signaling is disrupted by mutations in BMP ligands, receptors, or downstream signaling mediators, the proliferation and expansion of these mesenchymal cells are impaired. As a result, the palatal shelves may remain too small or misoriented, preventing them from coming into proper contact and fusing, which ultimately leads to a cleft palate and disruption of bone formation [31], [32]. Previous studies have demonstrated the expression of BMPs, BMP receptor 1a, and BMP antagonists in the developing palate, highlighting their critical roles in mouse palatogenesis across various developmental stages [33], [34], [35], [36]. Consistent with these findings, our study reveals distinct BMP signaling patterns within epithelial cells and the oral epithelium of the secondary palate at different embryonic stages. This signaling network is primarily mediated by two key ligand-receptor interactions [37]. Notably, Bmp7, a well-characterized BMP ligand, has been implicated in palatal development [37], [38], [39]. Our results reveal that Bmp7 expression peaks at E12.5 before gradually declining in later stages, underscoring its critical role during early palatogenesis. Similarly, BMP signaling activity is highest at E12.5 and E13.5 and then steadily decreases by E14.5. This temporal pattern aligns well with previous findings showing that Bmp4 is widely expressed throughout the palatal shelf at E13.5 but becomes confined to the mesenchymal layer beneath the oral epithelium by E14.5 [40]. These findings reinforce the dynamic role of BMP signaling in coordinating secondary palate development and underscore its tightly regulated spatial and temporal activity during craniofacial morphogenesis.

Recent work using spatiotemporal scRNA-seq combined with stereo-seq has further highlighted the cellular complexity of palate development [41]. That study resolved both mesenchymal and epithelial subsets into spatially distinct domains, identifying mesenchymal subpopulations linked to ossification and tissue remodeling, as well as epithelial programs enriched for immune responses. Importantly, signals such as Tgfβ3 and Pthlh were localized to the midline epithelial seam, implicating them in epithelial-mesenchymal transition. Together with our findings, these complementary datasets underscore the value of multiomic and spatial approaches in dissecting intercellular signaling dynamics and provide convergent insights into the mechanisms underlying secondary palate morphogenesis.

Our findings reveal a pronounced activation of BMP signaling in the oral epithelium at E12.5, suggesting a critical role in palatal development, particularly at the rugae. This observation aligns with previous studies indicating that Bmp4 induces the expression of sonic hedgehog (SHH) within the oral epithelium at the rugae during palatogenesis [37], [39], [42]. Moreover, a concurrent decline in both Bmp4 and SHH signaling has been reported in the developing palate at E13.5 [43]. At E12.5, the likelihood of communication between the oral epithelium and adjacent mesenchymal populations is notably high, as evidenced by the elevated expression of Bmp7. This gene is predominantly expressed in both epithelial and mesenchymal cells across various orofacial structures, including the peripheral regions of the palatal shelves. Notably, Bmp7-deficient mice have been shown to develop cleft palate, underscoring its essential role in palatogenesis [31], [44], [45]. During early embryonic development, Bmp7 functions as an inhibitor of epithelial-mesenchymal transition (EMT) induced by TGFβ. This is supported by its high expression levels in the oral epithelium at E12.5, followed by a progressive decline in subsequent stages, as demonstrated in our study. These findings are consistent with previous research demonstrating that Bmp7 suppresses TGFβ2-induced EMT in the lens epithelium, thereby preventing cataract formation [46]. Collectively, our results highlight the dynamic regulatory role of BMP signaling in coordinating epithelial-mesenchymal interactions during palatal development.

This study proposes a model in which the PDGF signaling pathway plays a critical role in mediating epithelial-mesenchymal interactions, thereby regulating the proliferation and differentiation of specific epithelial cell populations, particularly the dental epithelium, during palatogenesis at E12.5. We hypothesize that epithelial-derived Pdgfa and Pdgfc ligands signal through Pdgfra and Pdgfrb receptors expressed in mesenchymal cells, orchestrating key developmental processes in secondary palate formation. PDGF signaling is essential for mesenchymal cell survival and migration, particularly in neural crest-derived mesenchyme, which contributes to palate formation. PDGF deficiency results in defective palatal rugae patterning and fusion failure [47], [48]. Notably, inhibition of the PDGF pathway has been shown to alter the proliferative responses of skeletal stem and progenitor cells while accelerating their differentiation toward bone regeneration [49], [50]. Upon ligand binding, PDGF receptors undergo tyrosine phosphorylation within their cytoplasmic domains, initiating downstream signaling cascades. These molecular events regulate critical cellular functions, including proliferation, migration, extracellular matrix deposition, and the initiation of epithelial-to-mesenchymal transition, underscoring the multifaceted role of PDGF signaling in craniofacial development [51]. Our results highlight correlative patterns of PDGF and BMP signaling during secondary palate development, consistent with prior reports linking dysregulation of these pathways to orofacial clefts [52], [53], [54]. However, these associations remain associative, not causal, and future functional studies will be required to establish direct mechanistic roles.

Our study provides novel insights into the signaling pathways governing the directional movement of secondary palate cells across distinct developmental stages, with a particular focus on the receptor genes *Pdgfr-a* and *Pdgfr-b* and the ligand genes *Pdgfa* and *Pdgfc*. These genes exhibit critical signaling interactions in the dental epithelium at E12.5, with their activity diminishing by E14.5. Our findings suggest that PDGF signaling orchestrates epithelial cell growth, migration, and differentiation within the palatal shelves during E12.5. This is consistent with previous reports indicating that *Pdgfa* and *Pdgfc* are distinctly expressed in the epithelial lining of the oral and nasal cavities [55], [56], [57]. Notably, *Pdgfc* exhibits more pronounced signaling interactions at E12.5, corroborating studies showing that *Pdgfc* mutant embryos display cleft secondary palate and impaired palatal bone extension across the oronasal cavity [55].

Furthermore, our results underscore the integral role of *Pdgfa* and *Pdgfc* in the PDGFRα/PDGFRβ signaling network, with distinct contributions to palatogenesis and integumentary tissue morphogenesis. *Pdgfr-a* and *Pdgfa* are prominently expressed in CNC-derived odontogenic mesenchyme and dental epithelium, respectively, at all stages of tooth development. This consistent expression pattern underscores the paracrine role of PDGF signaling in regulating odontogenesis [58]. Consistent with this, our study reveals sustained expression of *Pdgfr-a* and *Pdgfa* in CNC-progenitor mesenchymal cells and dental epithelium, particularly at E12.5, followed by a progressive decline in later developmental stages. Supporting these findings, a previous study has demonstrated that cleft palate formation in *Pdgfr-a* mutant mice is associated with defects in the extracellular matrix of CNC-derived palatal mesenchymal [58]. Our bioinformatics analysis further suggests that interactions between *Pdgfr-a* and *Pdgfa*, as well as *Pdgfr-a* and *Pdgfc*, play pivotal roles in mediating epithelial-mesenchymal crosstalk essential for tooth and palate morphogenesis. These findings align with prior research demonstrating that disruption of *Pdgfr-a* signaling leads to anomalies in dental cusp growth and impairs the expansion of palatal shelves during craniofacial development [58].

In summary, our study offers a comprehensive understanding of the molecular mechanisms that regulate secondary palate development. By elucidating the dynamic interplay among WNT, BMP, and PDGF signaling pathways, we highlight their essential roles in orchestrating epithelial–mesenchymal interactions. Using single-cell multiome sequencing, we identified a peak in WNT, BMP, and PDGF signaling activity at E12.5, highlighting their crucial involvement in regulating cell proliferation, migration, and differentiation within the palatal shelves. WNT signaling, particularly through *Wnt4* and *Wnt6*, plays a key role in maintaining epithelial integrity and facilitating palatal shelf elevation, while BMP signaling, mediated by *Bmp4* and *Bmp7*, orchestrates mesenchymal proliferation and epithelial-mesenchymal transition. Additionally, PDGF signaling is essential for mesenchymal cell survival and the regulation of craniofacial morphogenesis, as demonstrated by the expression of *Pdgfra* and *Pdgfrb* in CNC-derived mesenchyme. The observed decline in these signaling pathways by E14.5 underscores the tightly regulated temporal dynamics required for proper palatogenesis. Furthermore, our findings suggest that disruptions in these pathways contribute to congenital craniofacial anomalies such as cleft lip and palate. Collectively, these results enhance our understanding of the intricate signaling networks underlying palate formation and provide a foundation for future studies on therapeutic interventions for craniofacial disorders. Furthermore, the integration of future spatial transcriptomics studies will be critical for advancing our understanding of secondary palate development. Spatially resolved data will allow us to precisely map gene expression patterns to the complex tissue architecture of the developing palate, dictating cell–cell interactions and signaling gradients that cannot be captured by bulk or single-cell transcriptomics alone. This spatial dimension will not only strengthen the biological relevance of our computational predictions but also help identify region-specific molecular programs that may underlie susceptibility to cleft palate. In this way, spatial transcriptomics will serve as a powerful next step to bridge our computational framework with experimental and translational research.

## 4 Methods

### 4.1 Single-cell multiome data processing

We obtained the single-cell data from Gene Expression Omnibus (GSE218576). We followed the procedures from previous work [14] to process the multiome data using the 10x Genomics Cell Ranger ARC pipeline (v2.0.0). Initially, the raw sequencing data were transformed into fastq format through 'cellranger-arc mkfastq'. Subsequently, the raw RNA-seq and ATAC-seq library files originating from the same sample were aligned to the UCSC mouse genome (mm10) and quantified via 'cellranger-arc count'. To standardize sequencing depth, sample aggregation was carried out using 'cellranger-arc aggr'.

The initial RNA count matrix and ATAC fragment data underwent additional processing using R packages Seurat (v4.0.3) by Hao et al. [15] for RNA data and Signac (v1.5.0) by Stuart et al. [59] for ATAC data. Filtering was applied based on specific criteria for each assay. For RNA data, cells with RNA counts ranging from 200 to 100,000 (200 <nCount_RNA < 100,000), cells with fewer than 7000 RNA features (nFeature_RNA < 7000), and cells with mitochondrial gene expression below 20 % (percent.mt < 20). For ATAC data, cells with ATAC counts between 200 and 100,000 (200 <nCount_ATAC < 100,000), cells with a nucleosome signal less than 2, and cells with a TSS enrichment greater than 1. After applying these filtering criteria, the dataset comprised 35,150 cells. The average sequencing depth was 73,631 reads per cell, resulting in an average detection of 2510 genes per cell.

Normalization of the gene expression count matrix was performed using single-cell transform (*SCT*). Principal Component (PC) analysis was carried out using the top 3000 highly variable features. To visualize the data in a Uniform Manifold Approximation and Projection (UMAP), the first 30 PCs were used as input.

For the scATAC data, peak calling was executed using the *MACS2* package [60] through the CallPeaks function within Signac (version 1.5.0). Each identified peak represents a potential cis-regulatory element (CRE). Subsequently, the CRE count matrix underwent normalization using Latent Semantic Indexing (LSI), which involves term-frequency (TF) inverse-document frequency (IDF) and Singular Value Decomposition (SVD). The first LSI component was not used in the downstream analysis due to its high correlation with sequencing depth.

Gene activity was quantified using the *GeneActivity* function within Signac (version 1.5.0). This process involved aggregating chromatin accessibility data that intersected with both the gene body and promoter regions of the genes.

### 4.2 CRE-gene linkage analysis

To establish connections between CREs and genes, we employed the LinkPeaks function within Signac [59], utilizing an approach originally described by SHARE-seq [61]. This involved calculating the Pearson correlation coefficient between gene expression and the accessibility of CREs. Only CREs located within a specified distance (default: 5 ×10^5^ base pairs) from the gene's transcription start site were considered in the model. To account for potential biases, covariates such as GC content, overall accessibility, and CRE size were integrated into the model. P-values were adjusted using the Benjamini-Hochberg method for multiple testing correction [62]. We retained only high-confidence CRE-gene links characterized by an adjusted p-value of less than 0.05 and coefficients greater than 0 for further downstream analysis.

### 4.3 Systematic inference of cell-cell communication

CellChat was employed to systematically deduce cell-cell communication patterns by integrating single-nuclear RNA sequencing (snRNA-seq) data with the pre-existing ligand-receptor interaction database (including mice), CellChatDB [17]. CellChat predicts potential cell-cell interactions in three steps. First, it identifies differentially over-expressed ligands and receptors within each cell group. Second, it calculates the communication probability between two cell groups based on the average expression levels of ligands and receptors, along with relevant cofactors. Third, it validates interactions using permutation tests to determine statistical significance. CellChat then generates an intercellular communication network for each ligand-receptor pair, with communication probabilities as edge weights. Additionally, it computes a signaling pathway-specific network by aggregating the communication probabilities of all associated ligand-receptor pairs. To ensure robustness, the algorithm automatically excluded clusters with insufficient cell numbers at a given stage, as very small populations can produce unstable or spurious communication inferences. This automated filtering step ensures that all communication networks reported are based on biologically meaningful and statistically supported interactions, while minimizing technical noise introduced by undersampled populations.

### 4.4 Quantitative assessment of cell-cell communication across different embryonic stages

To detect alterations in signaling between conditions, we used the comparative CellChat framework. This two-tiered approach first assesses global signaling changes at the cell population level and then examines dysregulated signaling pathways and ligand-receptor pairs. At the cell population level, we pinpoint specific populations with significant interaction changes and identify sources and targets of altered signaling across developmental stages. At the pathway and ligand-receptor pair level, we analyze detailed signaling changes, focusing on network architecture and interaction strength. This framework provides a concise overview of these analyses.

### 4.5 Fundamental principles of intercellular communication

We started with a broad perspective to uncover the fundamental principles governing cell–cell communication. In comparing these interactions across different developmental stages, our analysis addresses two key questions: (1) which cell type interactions undergo significant alterations, and (2) how do the primary sources and recipients of signals shift between conditions? To uncover changes in signaling sources and targets under different conditions, we employed network centrality analysis. This analysis involved quantifying the likelihood of a cell population serving as a signaling source (outdegree) and as a signaling target (indegree). Outdegree and indegree centralities were computed by aggregating the outgoing and incoming communication probabilities associated with each cell population across all signaling pathways. Next, we calculated the differential outdegree and indegree centralities for each cell population and represented them in a two-dimensional (2D) space. This visualization method enables the easy identification of cell populations that have undergone significant alterations in either sending or receiving signals between developing stages.

### 4.6 Recognition of enhanced and diminished ligand-receptor pairs

To identify statistically significant upregulated and downregulated ligand-receptor pairs when comparing developmental stages, we integrate cell-cell communication analysis with differential gene expression analysis. Specifically, for each cell group, we employ the *Wilcoxon* rank-sum test to compare gene expression between cells in the second stage and cells in the first stage. Signaling molecules are categorized as upregulated in the second stage if they meet the following criteria: (i) p-values are less than 0.05, (ii) log-fold changes exceed 0.25, and (iii) more than 25 % of cells in the second stage exhibit expression of these signaling molecules. Conversely, signaling molecules are considered downregulated in the second stage if they were upregulated in the first stage. Ligand-receptor pairs are deemed upregulated or downregulated when both ligands and receptors exhibit corresponding upregulation or downregulation. The intercellular communication facilitated by these perturbed ligand-receptor pairs is depicted using a chord diagram, and this visualization is generated using the ‘circlize’ R package [63].

### 4.7 Cell-cell communication analysis of four secondary palate embryonic stages

We applied CellChat individually to datasets of the four developmental stages of the secondary palate and then combined the resulting CellChat objects for comparative analysis. Shared cell-cell communication information across all stages emphasized the consistent results. We combined the sub-clustering results of mesenchymal (Apm, Ppm, Per, Int, Chon, CNC), endothelial, and epithelial cells into 8 groups (Fig. 2A). Similarly, epithelial cell sub-clustering (oral epith, dent epith, aboral epith, periderm, nasal epith, undiff epith) resulted in 13 groups in total (Supplementary Figure S4A). To infer communication between these groups, we calculated the average expression levels of signaling molecules using a 10 % truncated mean, excluding the top and bottom 10 % of cells based on expression levels.

### 4.8 Statistical analysis

Data are presented as mean ± standard deviation (SD) or as otherwise specified, with sample sizes provided in the results section and figure legends. For violin plot comparisons, a two-tailed Wilcoxon rank-sum test was conducted in R package. Percentage changes were assessed using the probability test function in R. Differential gene expression analysis between cell clusters was performed using the Wilcoxon rank-sum test in R, with a significance threshold of *p* < 0.05 applied to identify marker genes.

## Data and code availability

The single-cell multiome dataset was retrieved from the Gene Expression Omnibus (GEO) with accession number GSE218576. The additional dataset at embryonic stage E11.5 was retrieved from the GEO with accession number GSE132462. Since no new custom codes were generated for this study, we primarily utilized established bioinformatics tools, including Seurat, PANDO, CellChat, ggplot2, Shiny, etc., in our analyses.

## Limitation of the study

Our study is designed as a computational framework to generate hypotheses and provide novel insights into orofacial development and the causes of cleft palate development. While experimental validation is crucial for confirming biological relevance, our work serves as an essential first step in prioritizing key targets and pathways for future functional studies. Given the complexity and resource-intensive nature of generating appropriate animal models, our findings can guide experimentalists in designing targeted validations. Additionally, we have employed rigorous computational methodologies, including cross-validation with existing datasets and complementary analytical approaches, to enhance the robustness of our predictions.

## CRediT authorship contribution statement

**Fangfang Yan:** Methodology, Data curation. **Lukas M. Simon:** Writing – review & editing, Visualization. **Zhongming Zhao:** Writing – review & editing, Funding acquisition, Conceptualization. **Andi Liu:** Formal analysis. **Toshiyuki Itai:** Writing – original draft, Visualization, Formal analysis. **Yulin Dai:** Writing – review & editing, Writing – original draft, Software, Methodology, Investigation, Formal analysis. **Usama Hussein:** Writing – review & editing, Writing – original draft, Visualization, Software, Methodology, Formal analysis, Data curation.

## Declaration of Competing interest

The authors declare that they have no known competing financial interests or personal relationships that could have appeared to influence the work reported in this paper.
