# Supplementary material for: Single-cell multiome characterizing intercellular communication and intracellular regulation of epithelium and mesenchymal during secondary palate development in mice
Source: Comput Struct Biotechnol J. 2025 Sep 24;27:4290–303. doi: 10.1016/j.csbj.2025.09.031 (PMC12538024; doi:10.1016/j.csbj.2025.09.031)
Supplement: Supplementary file 1 — Supplementary material [file mmc1.docx]

**Supplmentary Figure legends**

**Supplementary Figure S1**: Workflow of the multiome dataset of secondary palate development.

**Supplementary Figure S2:** Unsupervised clustering of the multiome dataset showing major and subcluster cell types. (A) UMAPs visualization showing major cell types on snRNA and snATAC sequencing. (B) UMAPs visualization showing subclusters of cell types for CNC-derived mesenchymal cells based on snRNA, snATAC sequencing and Weighted Nearest Neighbor (WNN). (C) Unsupervised clustering of scRNA dataset (N=7966 cells) from mouse at embryonic stage E11.5 showing mesenchyme, epithelium, endoderm major cell types.

**Supplementary Figure S3:** Detailed comparison signaling networks sent among cell groups across multiple developmental stages of the secondary palate. (A) Hierarchical plots depict the inferred intercellular communication network between CNC-derived mesenchymal subsets with ebdothelium and epithelial as whole population at different embryonic stages. (B,C) Hierarchical plots depict the inferred intercellular communication network between distinct epithelial (oral, nasal and dental epithelium) and other epithelial subsets (Aboral, Undifferentiated epithelum and periderm) with the other epithelial and CNC-derived mesenchymal cell types. Circle sizes are proportionate to the number of cells in each cell type, and edge width represents communication probability.

**Supplementary Figure S4:** Unsupervised clustering of both mesenchymal and epithelial subtypes. (A) Jointly projection and clustering of prominent cells from different developmental stages onto 2D space, categorized by their roles in sending or receiving signaling networks at different embryonic stages. Dot size corresponds to the number of expressed cells, and colors denote cell types. (B) Heatmap illustrating outgoing signaling pathways from different cell types across various developmental stages. The top bar plot indicates the proportion of cell types involved in sending signals to neighboring cells. The stars (*) signify dental, nasal and oral epithelial cells at E12.5 as primary signal sources compared to other developmental stages. The stacked bar plots flanking the heatmap correspond to the y-axis, representing distinct signaling pathways between cell types (x-axis).

**Supplementary Figure S5:** Contribution of WNT pathway in nasal epithelium. (A) Hierarchical plots show the intercellular communication network for WNT signaling at different embryonic stages. Circle sizes are proportionate to the number of cells in each cell type, and edge width represents communication probability. (B) Heatmap showing the relative contribution of each ligand-receptor pair to the overall communication network of the WNT signaling pathway, calculated as the ratio of the total communication probability of the inferred network of each L-R pair to that of the WNT signaling pathway. (C) Stacked bar and ‘circlize’ plots show the relative contribution of each ligand-receptor pair to the overall communication network of the WNT signaling pathway. (D) Violin plot displaying the expression level of Wnt4 across the different cell types. (E) The dot plot illustrates the relative communication probability of the contributing L-R pair from epithelial to neighboring mesenchymal subsets.

**Supplementary Figure S6:** Contribution of BMP pathway in oral epithelium. (A) Hierarchical plots show the intercellular communication network for BMP signaling at different embryonic stages. (B) Heatmap showing the relative contribution of each ligand-receptor pair to the overall communication network of the BMP signaling pathway, calculated as the ratio of the total communication probability of the inferred network of each L-R pair to that of the BMP signaling pathway. (C) Stacked bar and ‘circlize’ plots show the relative contribution of each ligand-receptor pair to the overall communication network of the BMP signaling pathway. (D) Violin plot displaying the expression level of Bmp7 across the different cell subsets. (E) The dot plot illustrates the high communication probability of the contributing Bmpr1b and Bmpr1a to Bmpr2 from epithelial to neighboring mesenchymal subsets.

**Supplementary Figure S7:** Contribution of PDGF pathway in dental epithelium. (A) Hierarchical plots show the intercellular communication network for PDGF signaling. (B) Heatmap showing the relative contribution of each ligand-receptor pair to the overall communication network of the PDGF signaling pathway, calculated as the ratio of the total communication probability of the inferred network of each L-R pair to that of the PDGF signaling pathway. (C) (Top) Stacked bar and (Bottom) circlize plot showing the relative contribution of each ligand-receptor pair to the overall communication network of the PDGF signaling pathway. (D) Violin plot displaying the expression level of Pdgfa and Pdgfc markers across the different subsets. (E) The dot plot illustrates the high communication probability of the contributing Pdgfc and Pdgfc to Pdgfra from epithelial to neighboring mesenchymal subsets.
