# Supplementary figures and images for: Single-cell multiome characterizing intercellular communication and intracellular regulation of epithelium and mesenchymal during secondary palate development in mice
Source: Comput Struct Biotechnol J. 2025 Sep 24;27:4290–303. doi: 10.1016/j.csbj.2025.09.031 (PMC12538024; doi:10.1016/j.csbj.2025.09.031)

## Supplementary Figure S1: Workflow of the data analysis

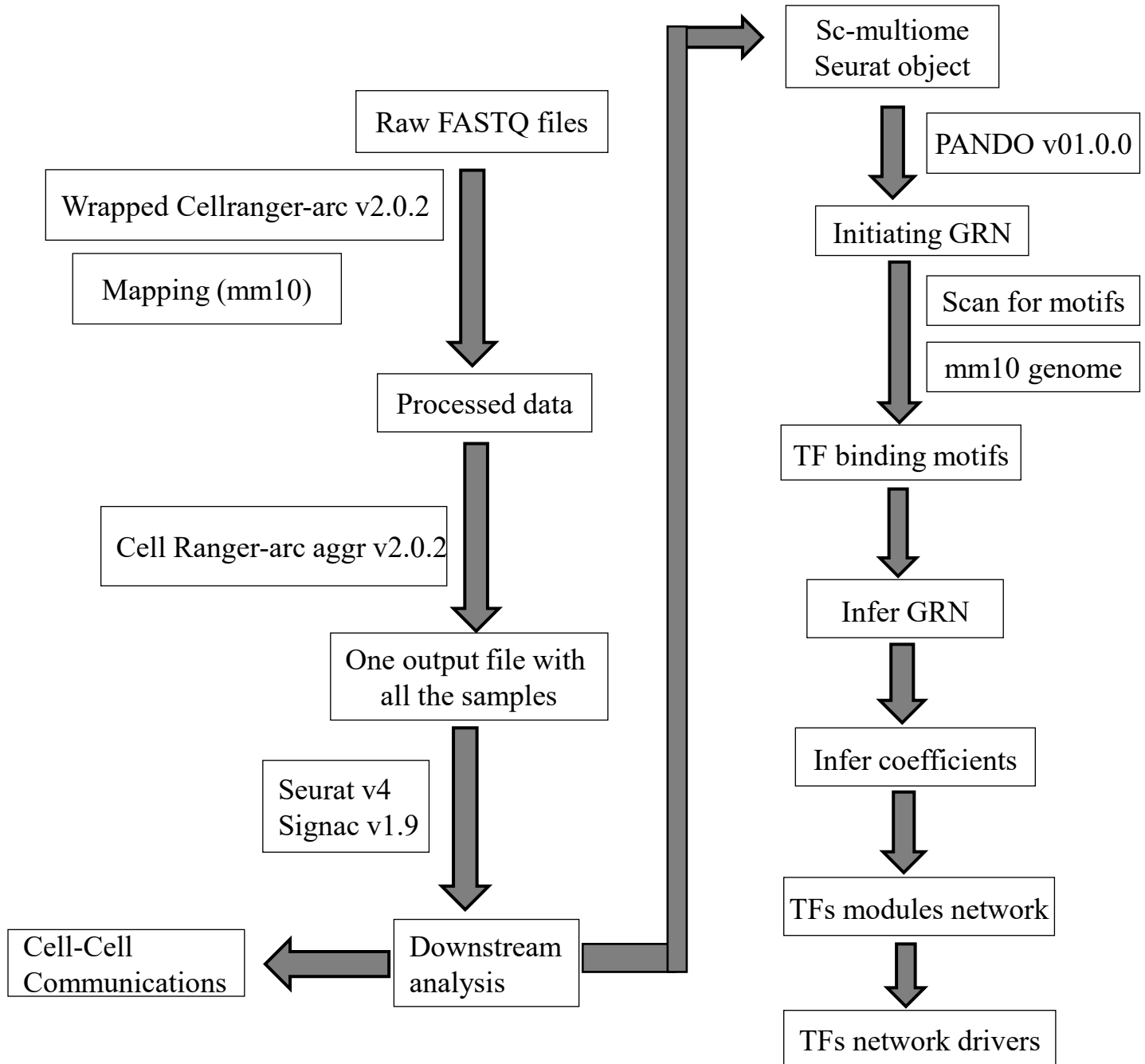

Supplement: Supplementary file 2 — Supplementary material [file mmc2.pdf]

Supplementary Figure S5: Contribution of WNT pathway in nasal epithelium

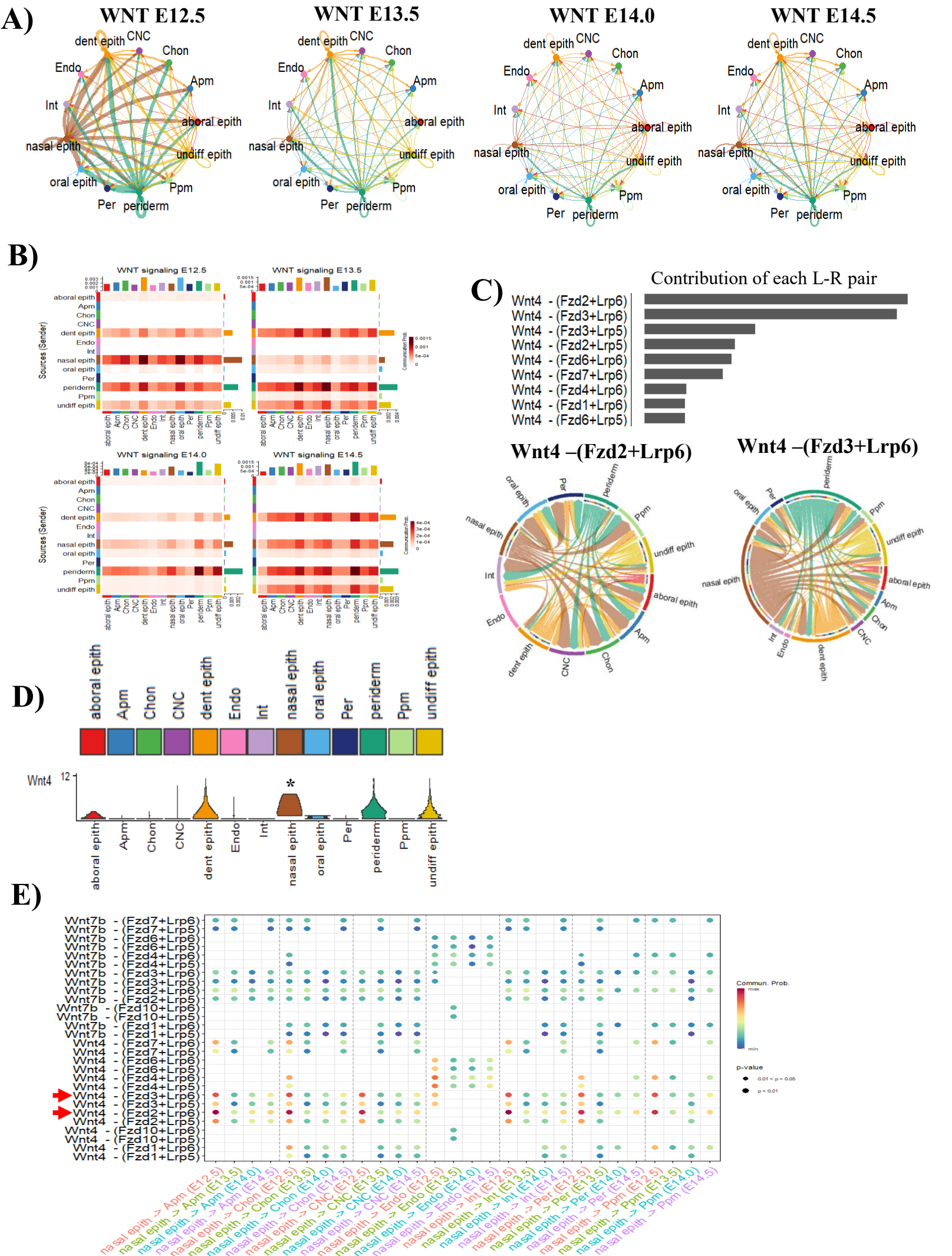

Supplement: Supplementary file 6 — Supplementary material [file mmc6.pdf]

Supplementary Figure S6: Contribution of BMP pathway in oral epithelium

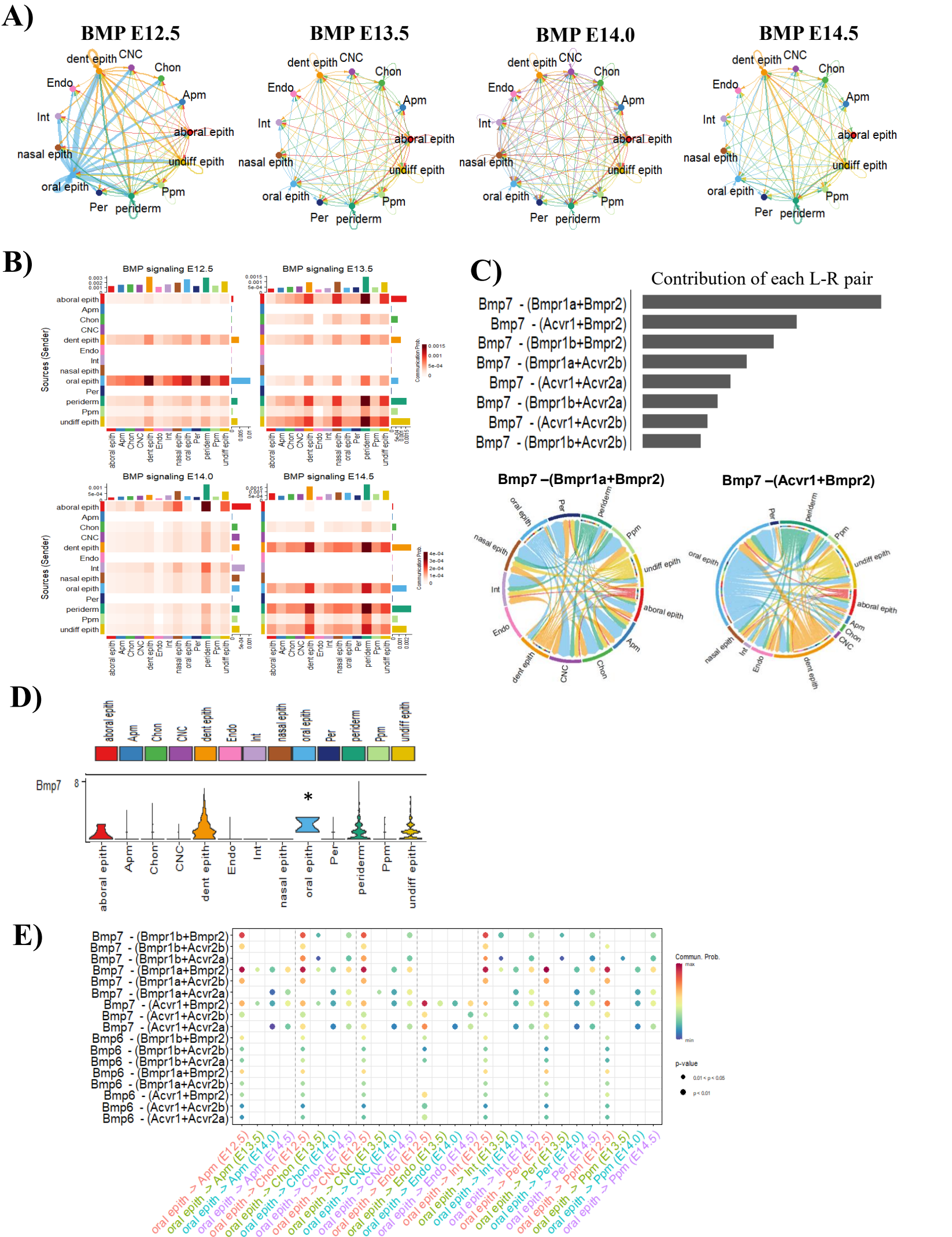

Supplement: Supplementary file 7 — Supplementary material [file mmc7.pdf]

Supplementary Figure S7: Contribution of PDGF pathway in dental epithelium.

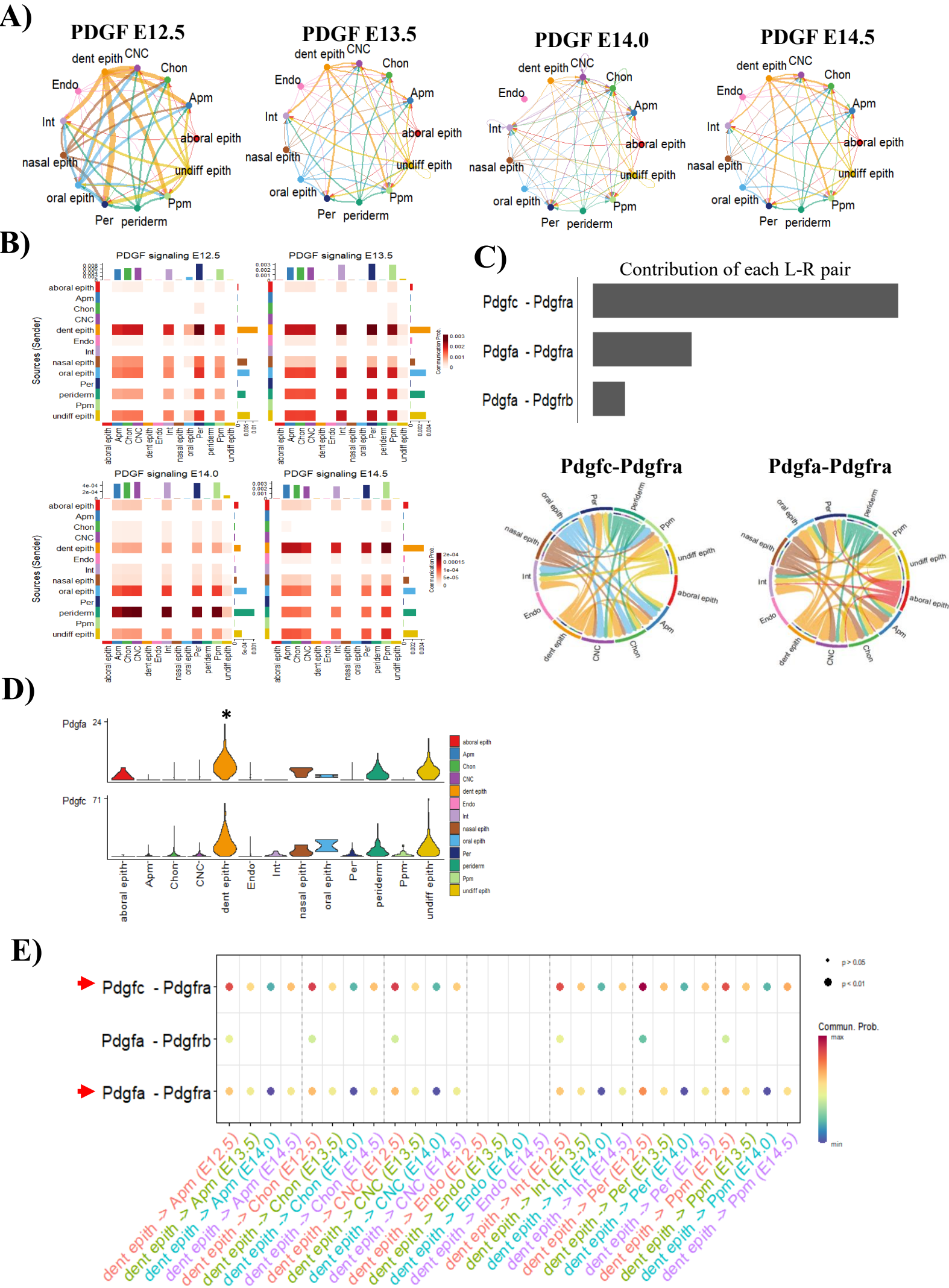

Supplement: Supplementary file 8 — Supplementary material [file mmc8.pdf]
