# Supplementary material for: Single-cell multiome characterizing intercellular communication and intracellular regulation of epithelium and mesenchymal during secondary palate development in mice
Source: Comput Struct Biotechnol J. 2025 Sep 24;27:4290–303. doi: 10.1016/j.csbj.2025.09.031 (PMC12538024; doi:10.1016/j.csbj.2025.09.031)
Supplement: Supplementary file 3 — Supplementary material [file mmc3.pdf]

Supplementary Figure S2: Unsupervised clustering of the multiome dataset and mouse E11.5 dataset showing major and subcluster cell types.

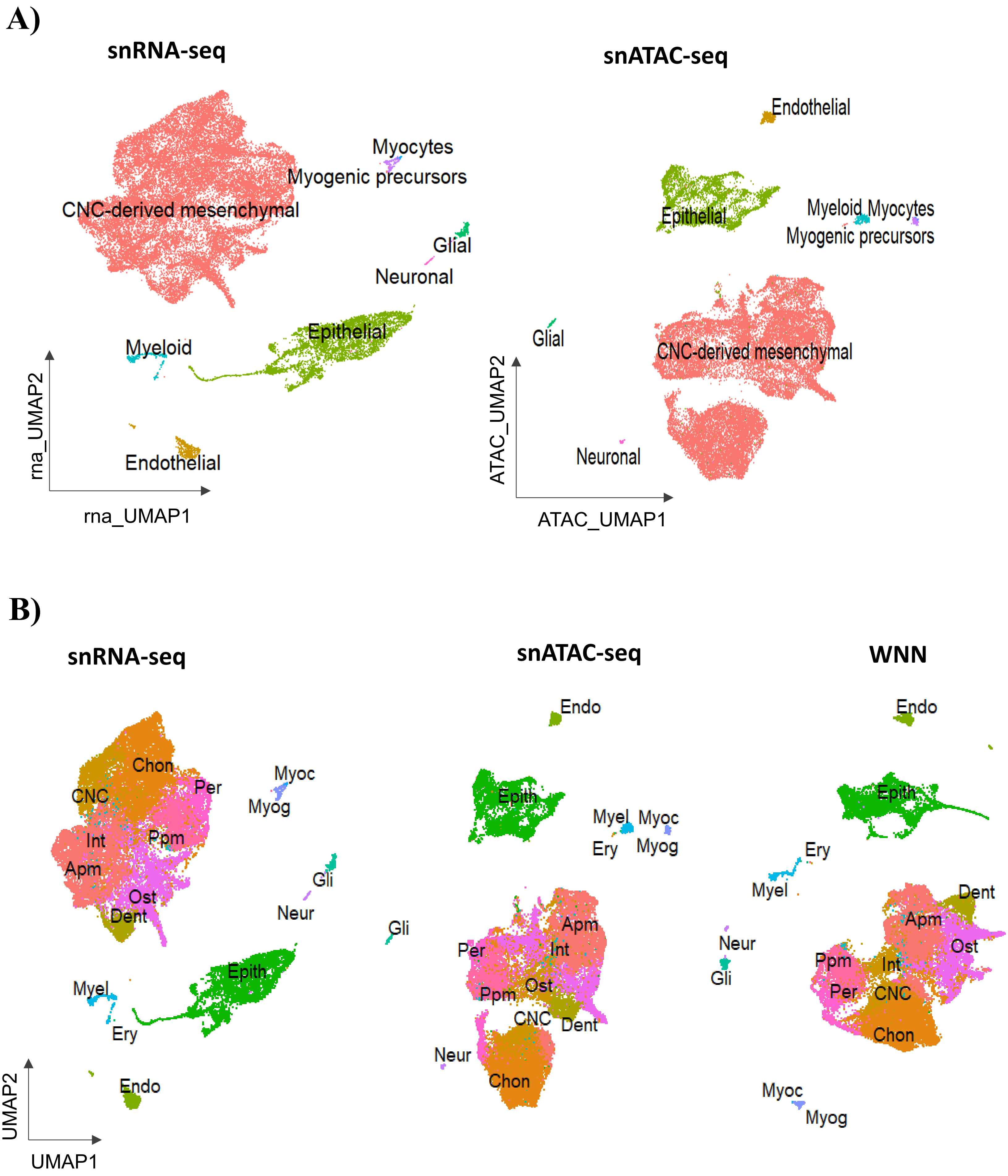

C)

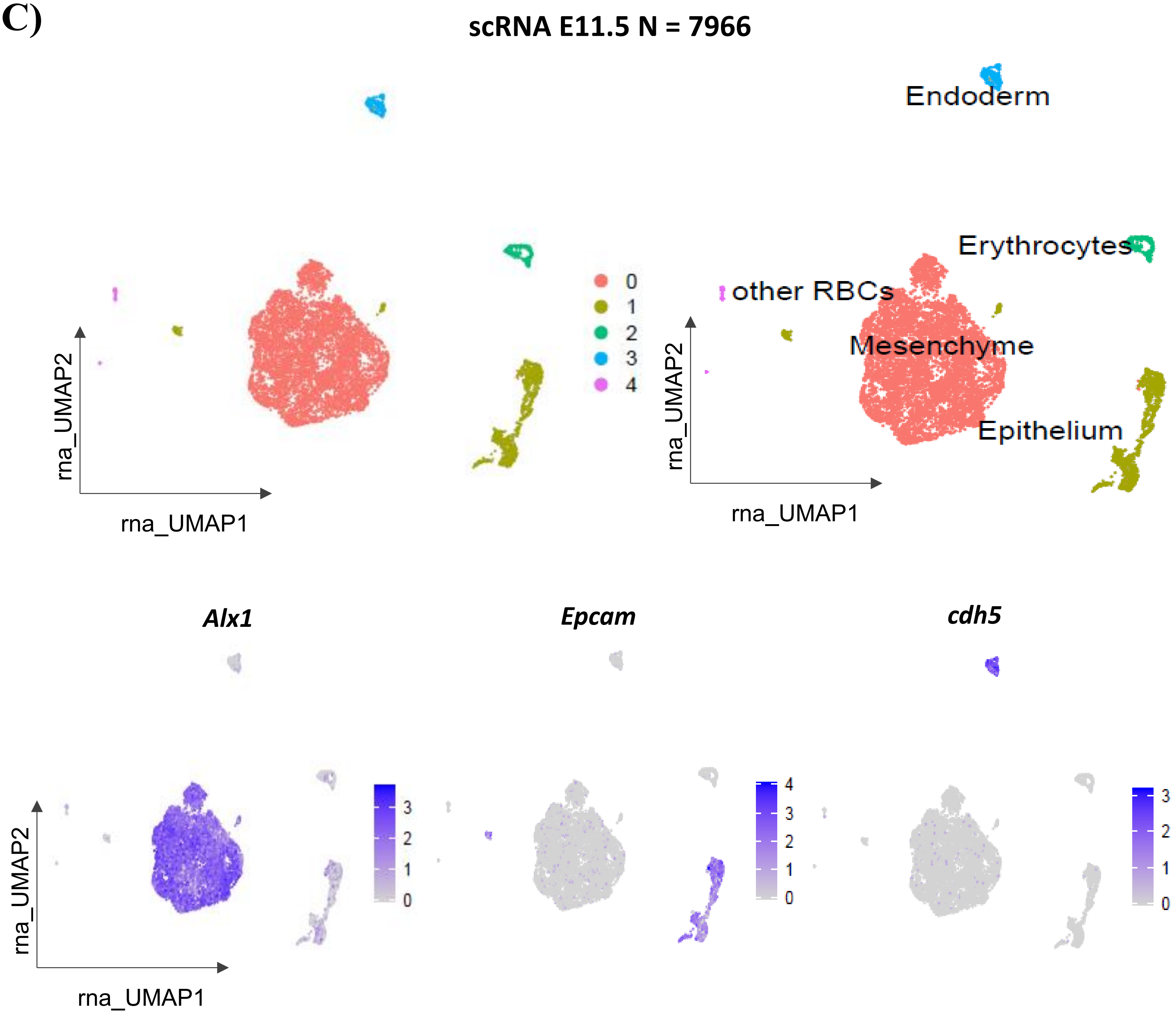
scRNA E11.5 N = 7966
0
1
2
3
4
rna_UMAP2
rna_UMAP1
Endoderm
Erythrocytes
other RBCs
Mesenchyme
Epithelium
rna_UMAP2
rna_UMAP1
Alx1
Epcam
cdh5
3
2
1
0
4
3
2
1
0
3
2
1
0
rna_UMAP2
rna_UMAP1
