# Supplementary material for: Single-cell multiome characterizing intercellular communication and intracellular regulation of epithelium and mesenchymal during secondary palate development in mice
Source: Comput Struct Biotechnol J. 2025 Sep 24;27:4290–303. doi: 10.1016/j.csbj.2025.09.031 (PMC12538024; doi:10.1016/j.csbj.2025.09.031)
Supplement: Supplementary file 4 — Supplementary material [file mmc4.pdf]

Supplementary Figure S3. Detailed comparison signaling networks sent among cell groups across multiple developmental stages of secondary palate.

A)

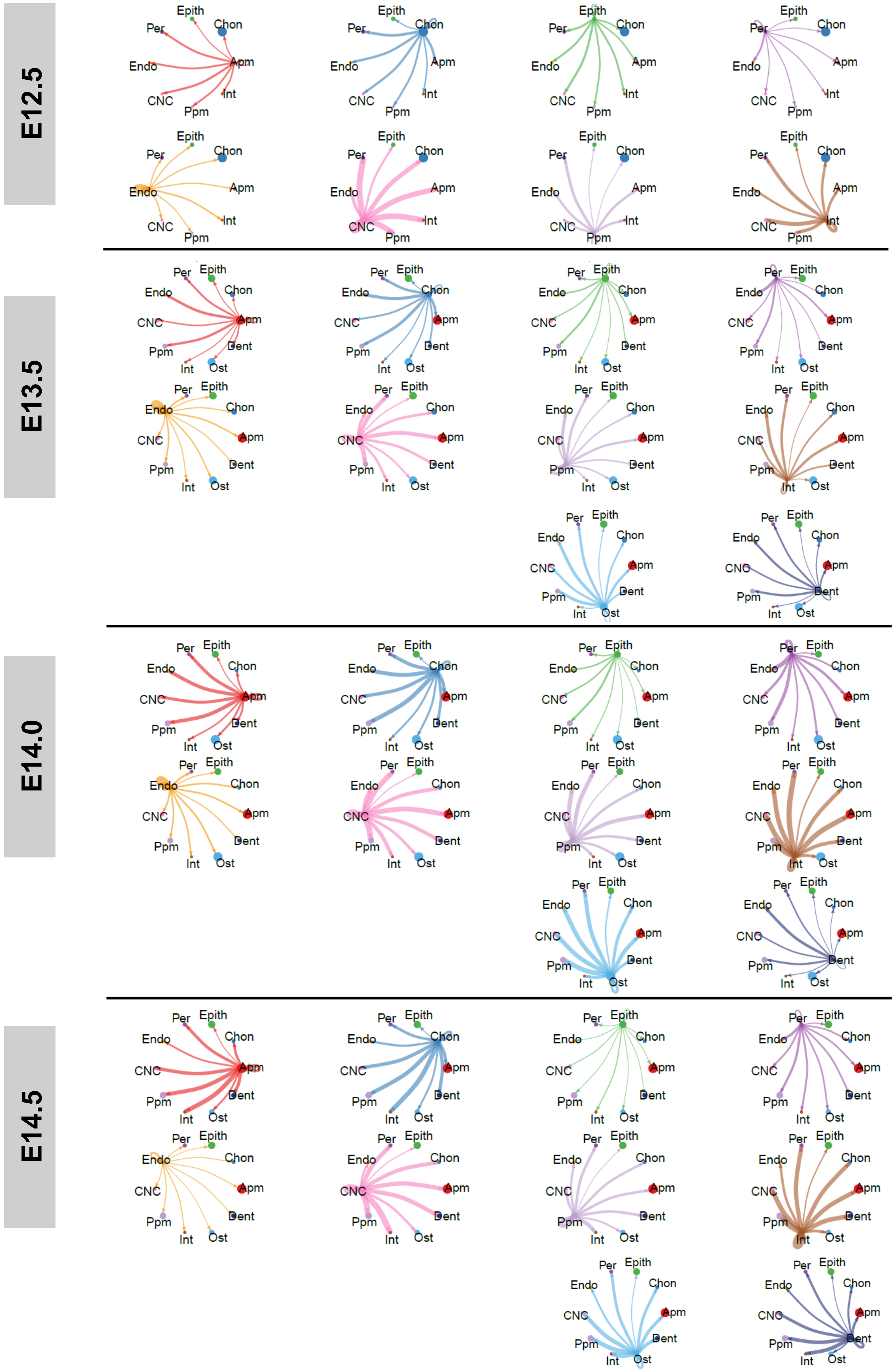

B)

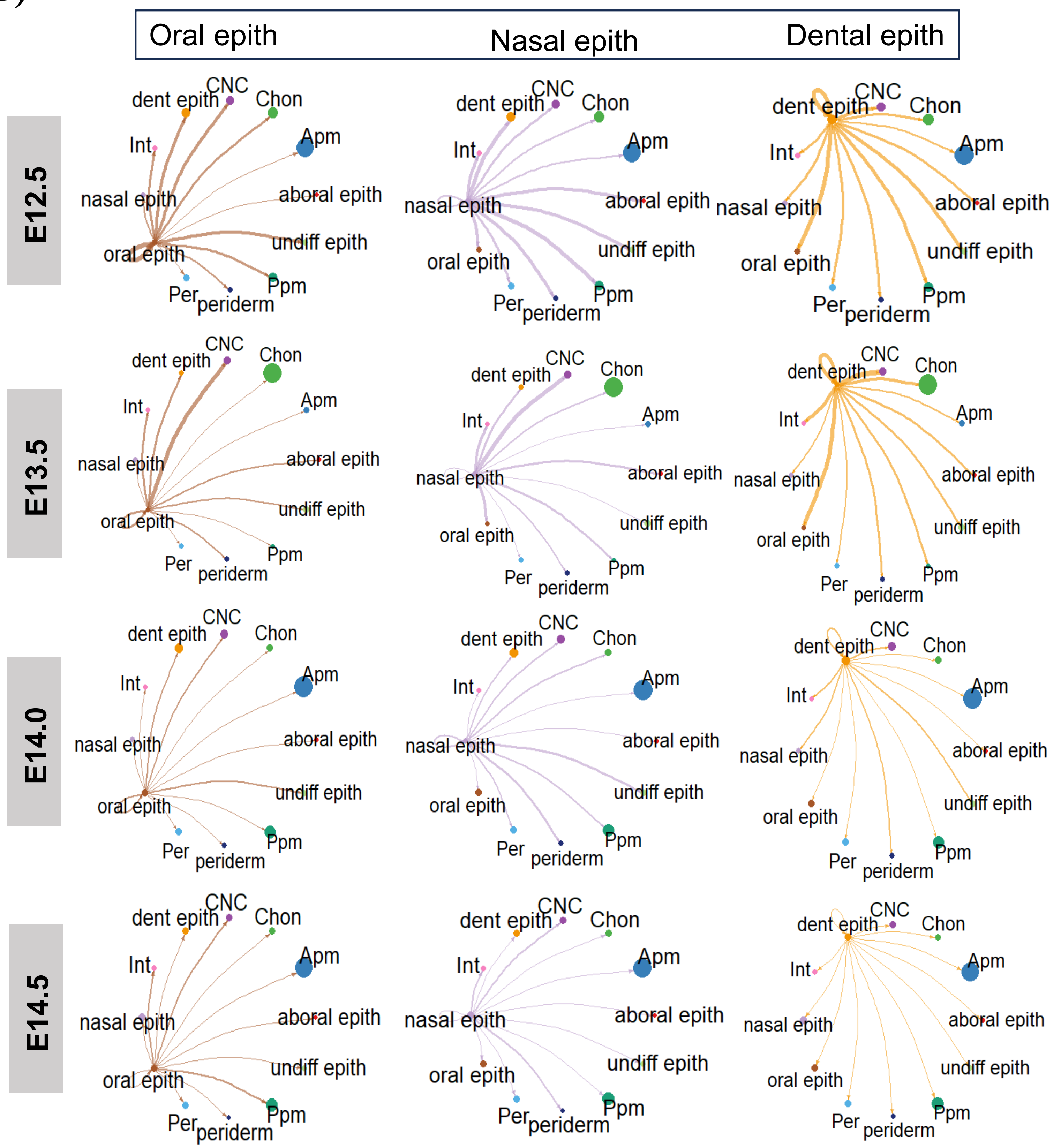

Oral epith
Nasal epith
Dental epith
E12.5
E13.5
E14.0
E14.5
dent epith
CNC
Chon
Int
Apm
nasal epith
aboral epith
oral epith
undiff epith
Per
periderm
Ppm

C)

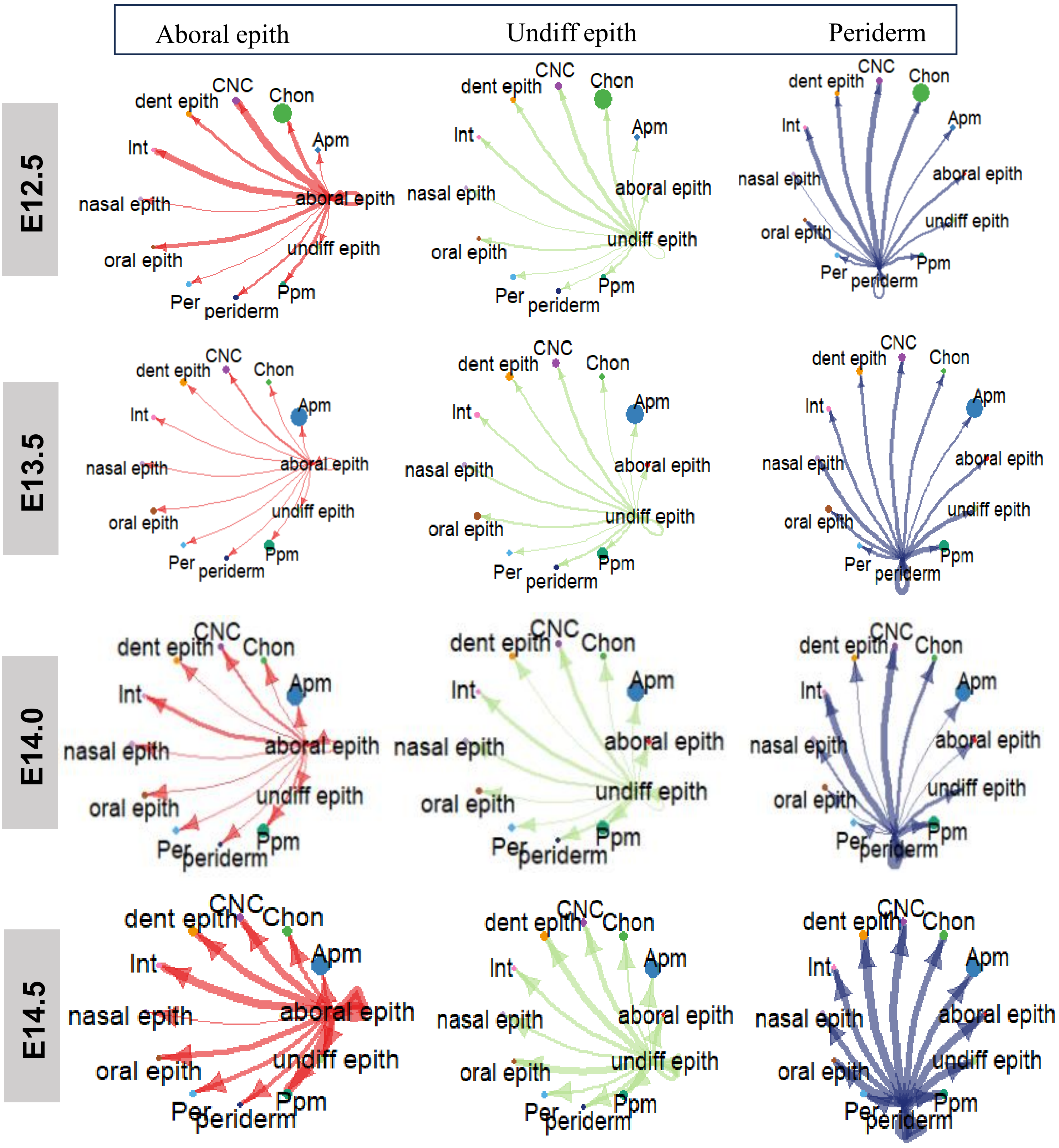
Aboral epith
Undiff epith
Periderm
E12.5
E13.5
E14.0
E14.5
dent epith
CNC
Chon
Int
Apm
nasal epith
aboral epith
oral epith
undiff epith
Per
periderm
Ppm
