# Supplementary material for: Single-cell multiome characterizing intercellular communication and intracellular regulation of epithelium and mesenchymal during secondary palate development in mice
Source: Comput Struct Biotechnol J. 2025 Sep 24;27:4290–303. doi: 10.1016/j.csbj.2025.09.031 (PMC12538024; doi:10.1016/j.csbj.2025.09.031)

Supplementary Figure S4. Unsupervised clustering of both mesenchymal and epithelial cell groups in 2D space.

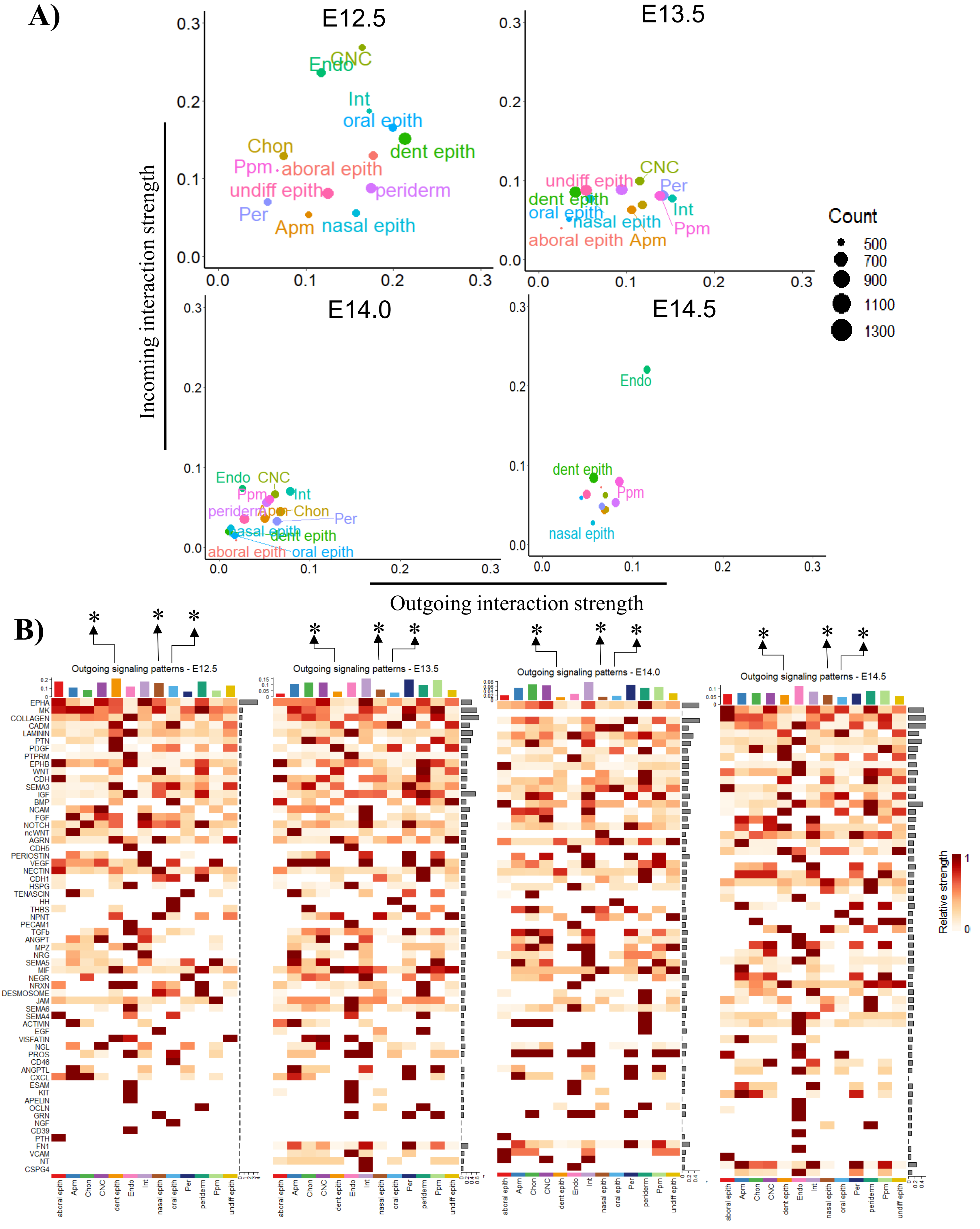

Supplement: Supplementary file 5 — Supplementary material [file mmc5.pdf]
